# Proteomic Analysis Reveals Differences in Tolerance to Acid Rain in Two Broad-Leaf Tree Species, *Liquidambar formosana* and *Schima superba*

**DOI:** 10.1371/journal.pone.0102532

**Published:** 2014-07-15

**Authors:** Juan Chen, Wen-Jun Hu, Chao Wang, Ting-Wu Liu, Annie Chalifour, Juan Chen, Zhi-Jun Shen, Xiang Liu, Wen-Hua Wang, Hai-Lei Zheng

**Affiliations:** 1 Key Laboratory of the Coastal and Wetland Ecosystems, Ministry of Education, College of the Environment and Ecology, Xiamen University, Xiamen, Fujian, China; 2 Institute of Urban and Environment, Chinese Academy of Sciences, Xiamen, P.R. China; 3 Department of Biology, Huaiyin Normal University, Huaian, Jiangsu, P.R. China; 4 Department of Biology and Chemistry, City University of Hong Kong, Kowloon, Hong Kong, SAR, China; Institute of Botany, Chinese Academy of Sciences, China

## Abstract

Acid rain (AR) is a serious environmental issue inducing harmful impacts on plant growth and development. It has been reported that *Liquidambar formosana*, considered as an AR-sensitive tree species, was largely injured by AR, compared with *Schima superba*, an AR-tolerant tree species. To clarify the different responses of these two species to AR, a comparative proteomic analysis was conducted in this study. More than 1000 protein spots were reproducibly detected on two-dimensional electrophoresis gels. Among them, 74 protein spots from *L. formosana* gels and 34 protein spots from *S. superba* gels showed significant changes in their abundances under AR stress. In both *L. formosana* and *S. superba*, the majority proteins with more than 2 fold changes were involved in photosynthesis and energy production, followed by material metabolism, stress and defense, transcription, post-translational and modification, and signal transduction. In contrast with *L. formosana*, no hormone response-related protein was found in *S. superba*. Moreover, the changes of proteins involved in photosynthesis, starch synthesis, and translation were distinctly different between *L. formosana* and *S. superba*. Protein expression analysis of three proteins (ribulose-1,5-bisphosphate carboxylase/oxygenase large subunit, ascorbate peroxidase and glutathione-S-transferase) by Western blot was well correlated with the results of proteomics. In conclusion, our study provides new insights into AR stress responses in woody plants and clarifies the differences in strategies to cope with AR between *L. formosana* and *S. superba*.

## Introduction

Acid rain (AR) emerged as a serious environmental issue as a consequence of the increasing industrial activities throughout the world [1]. Forty percent of the territory in China is seriously affected by AR since the late 1970s, especially in southern China [2]. The harmful impacts of AR on plants are observed in a wide array of biological processes. AR decreases seed germination [3], strips the protective wax from leaves [4], induces visible injury symptoms [1], disturbs plant nitrogen metabolism [5]. AR also decreases chlorophyll content and photosynthetic efficiency [3], [6], increases reactive oxygen species (ROS) production [7], accelerates the leaching of nutrients from plant foliage [8], which further inhibits tree radial growth, vertical growth and total tree biomass [4], [9].

*Liquidambar formosana* and *Schima superba* are both dominant broad-leaf tree species and are distributed over large surface areas in the forest of southern China [10]. Some field observations and laboratory experiments reported that, when compared with *S. superba*, *L. formosana* was largely injured by AR during the past decade, which had negative impacts on forest ecosystem [9]. Our previous study also found that AR more easily affected some physiological parameters in *L. formosana* seedlings than *S. superba*’s seedlings, e.g., seed germination, seedling growth, photosynthesis, antioxidant system, etc. [3], [11]. Thus, *L. formosana* is considered as an AR-sensitive species, while *S. superba* is an AR-tolerant species. Although the differential responses of *L. formosana* and *S. superba* to AR have been analyzed at the morphological and physiological level, a comprehensive elucidation of the molecular mechanisms underlying the different strategies to cope with AR between two tree species is still needed.

Proteomics is a powerful tool for providing new insights into complete proteomes at the organ, tissue and cell levels [12]. A number of proteomic analyses help us understand the molecular mechanisms of plants in responses to various environmental stresses including salinity [13], cold [14], heavy metal [15], etc. In our previous work, a wide array of proteins related to AR-resistance has been identified by comparative proteomic analysis in a model plant, *Arabidopsis thaliana* [5], [16]. However, little information is available in proteome analysis for tree species subjected to AR stress, and a comparison between AR-sensitive and AR-resistant broad-leaf tree species has not been fully conducted at the proteome level.

In the present study, we initiated a comparative proteomic study to systematically investigate the changes in protein profile in two broad-leaf tree species, *L. formosana* and *S. superba*, that are different sensitive to AR tolerance, when submitted to simulated AR (pH 3.0) treatment for one month. Based on two-dimensional electrophoresis (2-DE) and mass spectrometry (MS) analysis, a comprehensive inventory of proteins regulated by AR was established in the two tree species. The overall objectives of this study are (1) to provide valuable insights into AR stress responses in woody plants; (2) to clarify the differences in strategies to cope with AR between *L. formosana* and *S. superba*.

## Materials and Methods

### Plant materials and treatments

Seeds of *L. formosan*a and *S. superba* were purchased from Tree Seed Centre of Shuyang County in Jiangsu Province, China. The seeds have been mixed together when they were collected from independent individuals and families. Seeds were surface-sterilized with 0.5% hypochlorite for 30 min, then washed thoroughly with distilled water. Then the seeds were germinated in a soil/vermiculite (1∶1) mixture in an environmentally controlled growth chamber. For each species, three weeks old healthy seedlings with similar size were randomly transplanted into individual pots, each with a dimension of 24 cm (open top) × 13 cm (height) × 15 cm (flat bottom), and filled with soil/vermiculite (1∶1) mixture. Fifteen seedlings were planted in one pot. All seedlings were cultivated in the same controlled growth chamber with a daily temperature regime of 28/25°C (day/night), relative humidity of 60–70% and a 12-h photoperiod at 210 µmol m^−2^ s^−1^ photosynthetically active radiation (PAR). Three months later, the seedlings were divided into control group (CK) and simulated AR treatment group (AR). Each group had at least three replicates. The control group and simulated AR treatment group were sprayed once per day with the control (pH 5.6) solution and AR (pH 3.0) solution, respectively. The ion compositions of the control solution was adapted from Fan and Wang [4], AR solution was made from control solution and the pH was adjusted by adding a mixture of H_2_SO_4_ and HNO_3_ in the ratio of 5∶1. The final concentration of H_2_SO_4_ and HNO_3_ were 0.45 and 0.09 mM, respectively, which represents the average ion composition of rainfall in southern China [4]. After one month of treatment, a portion of fresh leaves was used for measuring some physiological parameters such as necrosis percentage, chlorophyll content, net photosynthetic rate, H_2_O_2_ content and so on. The remaining leaves were stored at −80°C for proteomic and Western blot analysis.

### Necrosis percentage and chlorophyll content measurements

At least 30 fully expanded leaves of each species were randomly selected from control and AR treatment groups and photographed using a digital camera. The necrosis percentage was calculated as described previously [16]. Chlorophyll was extracted from approximately 0.1 g fresh leaf slices directly into 10 ml ice-cold acetone (80%, v/v). The chlorophyll content (mg g^−1^ fresh weight (FW)) was determined as described previously [17].

### Net photosynthetic rate and chlorophyll fluorescence measurements

Three seedlings per species were randomly chosen from different pots and at least two fully emerged leaves per plant were selected for net photosynthetic rate (Pn) and chlorophyll fluorescence measurements. Pn was performed with a portable photosynthesis system (LI-6400, Li-Cor Inc., Lincoln, Nebraska, USA), as described previously [18]. According to the method of Liu et al [16], leaf chlorophyll fluorescence was measured using a pluse-amplitude-modulation fluorometer (PAM-2100, Heinz Walz, Effeltrich, Germany).

### Measurement of proline, malondialdehyde (MDA) and ROS production

Proline content was measured according to the method of Jiang et al [19]. The level of lipid peroxidation was measured by estimating MDA content using thiobarbituric acid (TBA) reaction [20]. Superoxide radical (O_2_^•-^) and H_2_O_2_ content was measured following the method of Chen et al [11].

### Protein extraction

Protein extraction was performed using phenol-based protocol described by Liu et al [5], with slight modifications. Briefly, frozen leaves (1.0 g) were ground with a mortar and pestle with liquid nitrogen, the ground powder was homogenized in pre-cooled extraction buffer (20 mM Tris-HCl, pH 7.5, 250 mM sucrose, 10 mM ethylene diamine tetraacetic acid (EDTA), 1 mM phenylmethyl-sulfonyl fluoride, 1 mM 1,4-dithiothreitol (DTT) and 1% Triton X-100) on ice. Then an equal volume of ice-cold Tris-HCl (pH 7.5) saturated phenol was added and the mixture was centrifuged (15,000 *g*, 4°C) for 15 min. The phenol phase was collected and proteins were precipitated with ammonium acetate in methanol for 10 h at −20°C. After centrifugation, the supernatant was discarded and the pellet was washed for three times using cold acetone containing 10 mM DTT. The washed protein pellets were dissolved in a lysis buffer (8 M urea, 2 M thiourea, 4% (w/v) 3-[(3-cholamidopropyl)dimethylammonio]-1-propane sulfonate (CHAPS), 1% (w/v) DTT and 0.5% (w/v) IPG buffer pH 4–7) at room temperature. The total protein concentration of the lysates was determined using a Bio-Rad protein assay reagent (Bio-Rad, Hercules, CA, USA).

### Two-dimensional electrophoresis, image and data analysis

Two-dimensional electrophoresis was conducted according to the methods of Bai et al [13] and Hu et al [21]. Isoelectric focusing (IEF) was done using an Ettan IPGphor system (GE Healthcare) PROTEAN electrophoresis system and immobilized IPG dry gel strips with a linear pH range (18 cm long, pH 4–7 linear) (GE Healthcare Amersham Bioscience, Little Chalfont, UK). Protein samples (800 µg) were loaded during the rehydration step at room temperature for 12 h. IEF was performed at 300 voltage (V), 500 V and 1,000 V for 1 h, a gradient to 8,000 V over 4 h, and kept at 8,000 V for a total of 80,000 volt-hours (Vh) at 20°C. Subsequently, focused strips were equilibrated in equilibration buffer as described by Yang et al [12]. For the second dimension, proteins were separated on 15% SDS polyacrylamide gels. Proteins spots were detected by staining the gels with Coomassie Brilliant Blue R-250. The 2-DE gels were scanned with a scanner (Uniscan M3600, China) and the gel images were analyzed with PDQuest software (Version 8.01, Bio-Rad, Hercules, CA), on the basis of their relative volume. Only those protein spots with significant (more than 2-fold change) and reproducible changes in three replicates were selected for next MS analysis.

### In-gel digestion, protein identification and classification analysis

The protein spots, which were differentially displayed under AR treatment, were excised from the preparative 2-D gels and digested by trypsin. After digestion, the peptide solution was collected and peptide mass fingerprint (PMF) was acquired using matrix-assisted laser desorption/ionization-time-of-flight mass spectrometry (MALDI-TOF MS) analysis (ReFlexTM III, Bruker, Bremen, Germany) as described previously [16]. The PMF spectra were used in online searches combined with the Mascot program search engine (http://www.matrixscience.com) and National Center for Biotechnology Information (NCBI) protein database (http://www.ncbi.nlm.nih.gov) (NCBInr, 17751536 entries, downloaded on April 17, 2012). PMF search parameters were set up as described previously [22]. Proteins with a MOWSE score >73 were considered as positive identifications. The identified proteins were searched with against the UniProt (http://www.uniprot.org) and/or NCBI protein database (http://www.ncbi.nlm.nih.gov) for updated annotation and homologous proteins identification. Afterwards, the successfully identified proteins were further classified using Functional Catalogue software (http://mips.gsf.de/projects/funcat).

### Western blot analysis

Western blot analysis was performed as described previously [11]. Total proteins (40 µg) extracted from *L. formosan*a and *S. superba* leaves were separated by 12% w/v standard sodium dodecyl sulfate polyacrylamide gel electrophoresis and then electrophoretically blotted to polyvinylidene difluoride membrane for 50 min. The membranes were blocked over-night with Western Blocking Buffer (TIANGEN, China). Protein blots were probed with primary antibodies of Rubisco large subunit (RuBISCO LSU) (AS03037-200, Agrisera, Sweden), ascorbate peroxidase (APX) (AS08368, Agrisera, Sweden) and glutathione-S-transferase (GST) (AS09479, Agrisera, Sweden), at dilution of 1∶5000, 1∶2000 and 1∶1000, respectively, for 4 h at room temperature with agitation. Next, the membranes were washed in phosphate buffered saline with Tween-20 solution (PBST) solution (50 mM Tris-HCl, pH 8.0, 150 mM NaCl, 0.05% Tween 20, v/v) three times and incubated with anti-rabbit IgG horseradish peroxidase (HRP) conjugated to alkaline phosphatase (Abcam, U.K., 1∶5000 dilution) for 1 h at room temperature to detect primary antibodies. *β*-actin (1∶5000, Santa Cruz, California, USA) was used as an internal control. After several washes with PBST solution, membranes were incubated in an enhanced chemiluminescence (ECL) substrate detection solution (TIANGEN, China) according to the manufacturer’s instructions. Images of protein blots were obtained using a CCD imager (FluorSMax Bio-Rad, Hercules, CA, USA). The optical density values of the protein signals were quantified using the Quantity One software (Bio-Rad, Hercules, CA, USA).

### Statistical analysis

For physiological measurements, at lease four independent repetitions were used. Values in figures and tables were expressed as means ± se. The statistical significance of the data was analyzed using a univariate analysis of variance (One-way ANOVA, Duncan’s multiple range test, *p*<0.05) with the SPSS 20.0 package (SPSS, Chicago, Illionis USA). For proteomic experiment, protein samples for 2-DE gel image analysis were extracted from three independent seedlings grown in three different pots in the same growth chamber. Thus, for each species, three independent biological replicates were performed in 2-DE gel image analysis. Statistic analysis for protein spot on 2-DE gels was performed using Student’s t-test (*p*<0.05) provided by PDQuest software as mentioned earlier.

## Results

### Morphological and physiological responses of *L. formosana* and *S. superba* to AR

**Figure 1:**
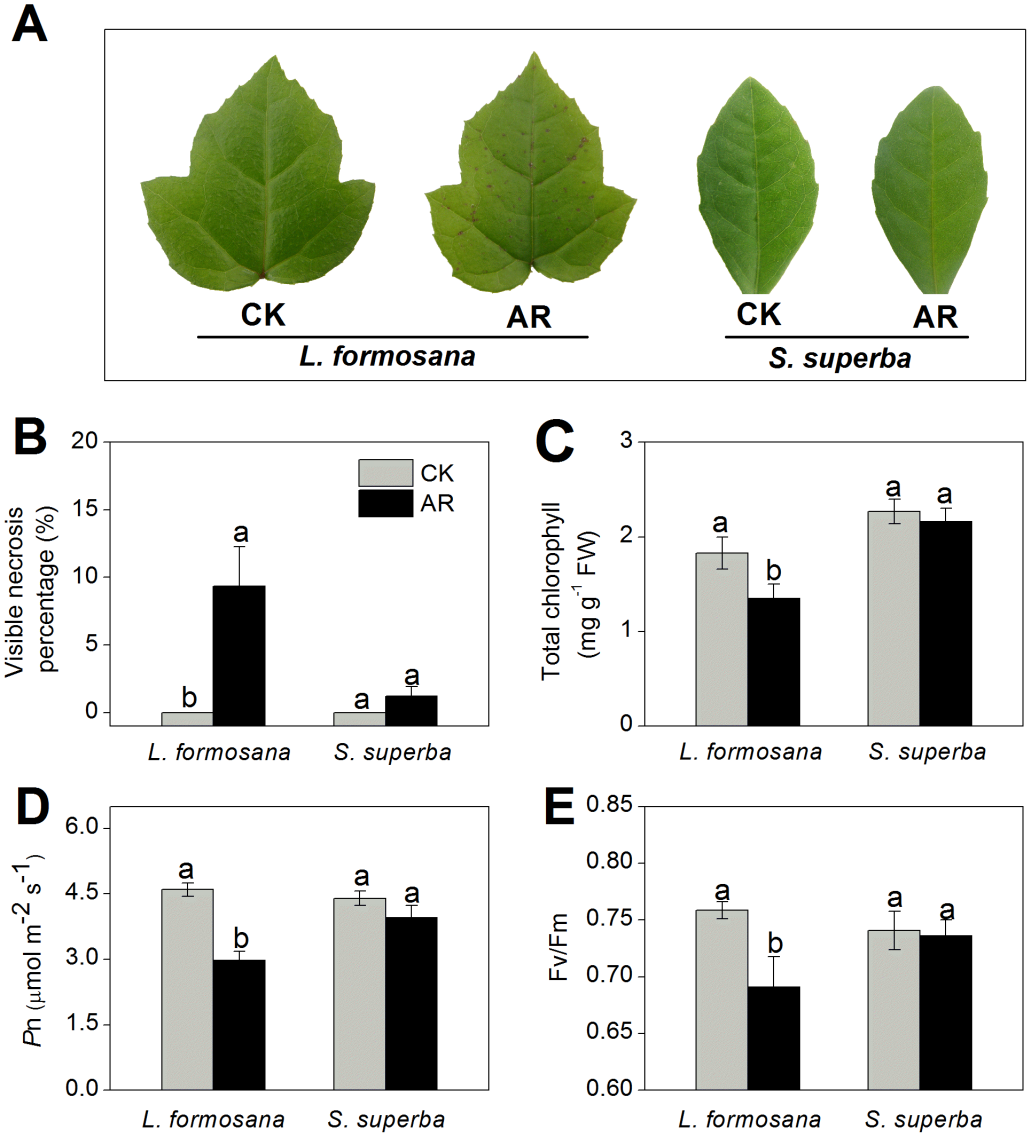
Effects of one-month AR on morphology and photosynthesis of *L. formosana* and *S. superba.* The pH of AR solution was adjusted to 3.0 by adding a mixture of H_2_SO_4_ and HNO_3_ in the ratio of 5∶1. The final concentration of H_2_SO_4_ and HNO_3_ were 0.45 and 0.09 mM, respectively. (A) Leaf injury phenotype. (B) Leaf necrosis percentage. (C) Total chlorophyll content. (D) Net photosynthetic rate (Pn). (E) Quantum efficiency of open PSII centers in a dark-adapted state (Fv/Fm). Columns labeled with different letters indicate significant differences at *p*<0.05.

**Figure 2:**
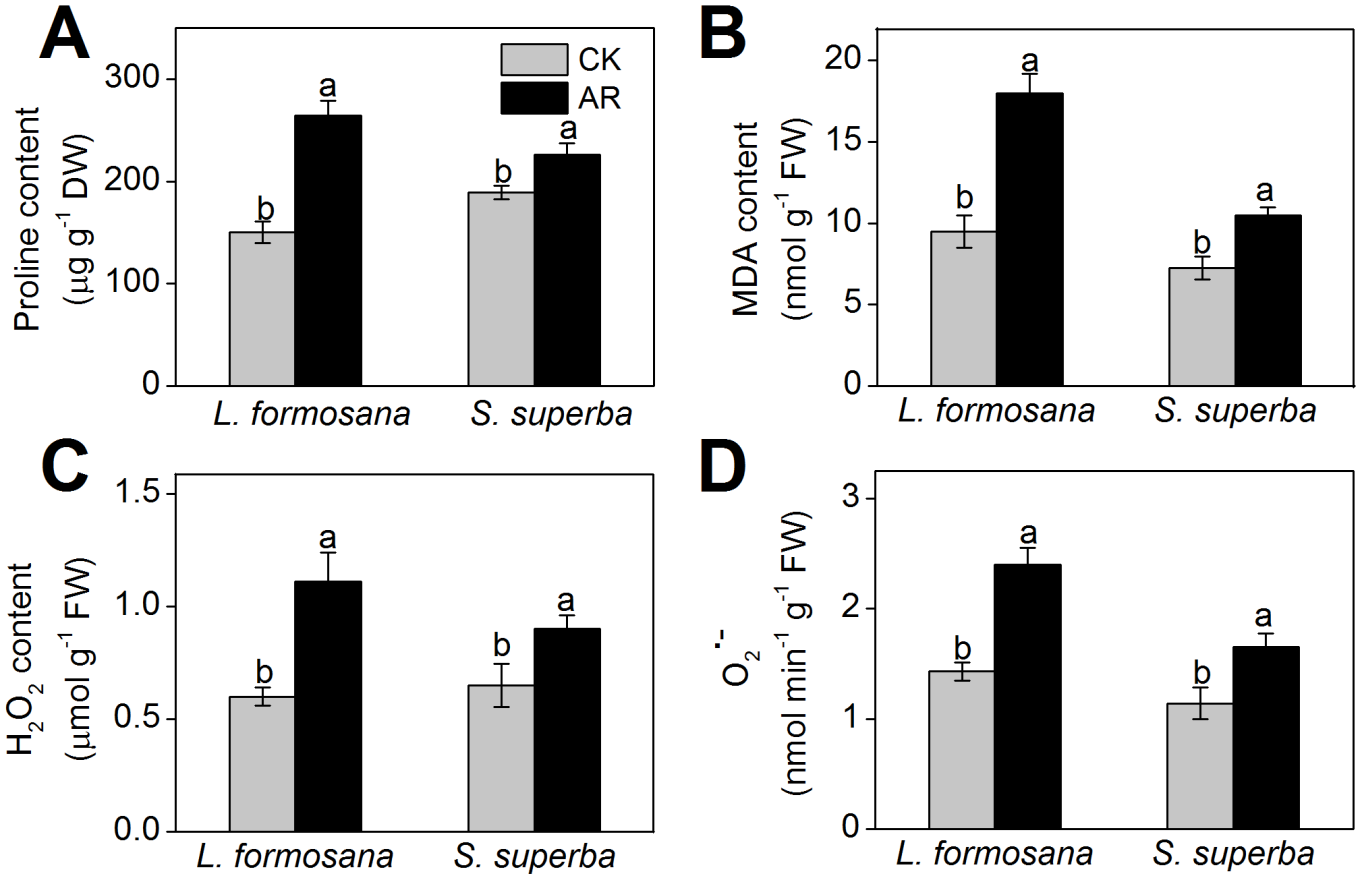
Changes in proline (A), MDA (B), H_2_O_2_ (C) and O_2_^•-^ (D) content after AR treatment. Columns labeled with different letters indicate significant differences at *p*<0.05.

As shown in Fig. 1A and B, remarkable yellowing symptom and significant necrosis emerge in *L. formosana* leaves after one-month exposure to AR. There was an important decrease in total chlorophyll content in AR-treated *L. formosana* leaves, however, no statistically significant change was found in *S. superba* (Fig. 1C). Pn and Fv/Fm in AR-treated *L. formosana* seedlings were remarkably inhibited, whereas AR slightly decreased Pn and Fv/Fm in *S. superba* (Fig. 1D and E). After AR treatment, proline content in *L. formosana* and *S. superba* increased by 76.0% and 19.7%, respectively, compared with the control (Fig. 2A). MDA contents in AR-treated *L. formosana* and *S. superba* increased by 89.4% and 44.8%, respectively (Fig. 2B). As shown in Fig. 2C and D, the levels of H_2_O_2_ and O_2_^•-^ in both *L. formosana* and *S. superba* were significantly stimulated by AR. In particular, compared with the control, H_2_O_2_ and O_2_^•-^ content increased by 83.3% and 67.8% in *L. formosana*, and by 38.4% and 44.7% in *S. superba*, respectively (Fig. 2C and D).

### Identification of AR-responsive proteins

**Figure 3:**
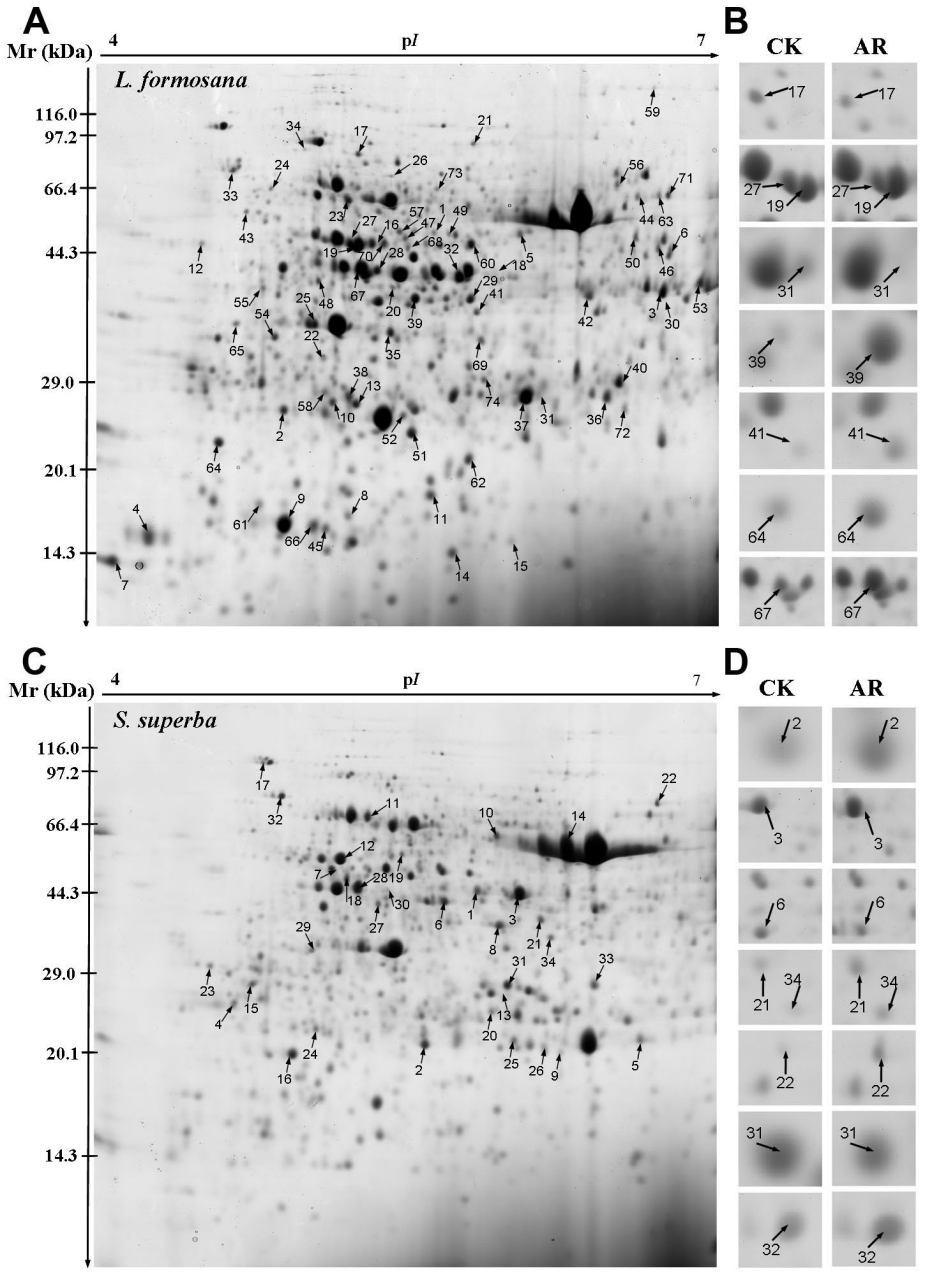
2D gel analysis of proteins extracted from *L. formosana* and *S. superba* leaves. The numbers assigned to the proteins spots correspond to those listed in Tables 1 and 2. (A) Representative 2-DE gels of *L. formosana* in which 74 spots showing at least 2-fold changes (*p*<0.05) under AR were identified by MALDI-TOF MS. (B) Close-up views of differentially expressed protein spots in *L. formosana* (highlighted by arrows). (C) Representative 2-DE gels of *S. superba* in which 34 spots showing at least 2-fold changes (*p*<0.05) under AR were identified by MALDI-TOF MS. (D) Close-up views of differentially expressed protein spots in *S. superba* (highlighted by arrows).

To investigate the differentially expressed proteins in *L. formosana* and *S. superba* exposed to AR treatment, comparative proteomic analysis was performed on Coomassie-stained 2-DE maps shown in Fig. 3. Over 1000 protein spots reproducibly separated and matched between control and AR gels, 74 protein spots in *L. formosana,* and only 34 protein spots in *S. superba* had at least a 2-fold greater abundance in either AR or control (Fig. 3). The identified proteins in *L. formosana* and *S. superba* by MALDI-TOF MS analysis are presented in Tables 1 and 2, respectively. Because the complete annotated sequences of *L. formosana* and *S. superba* genomes are not yet available, all identified proteins were functionally classified by UniProt and NCBI databases according to their homology with other proteins. Functional annotations in databases existed for the majority of the protein spots, while 12 proteins (spots L63–L74) in *L. formosana* and 6 proteins (spots S29–S34) in *S. superba* were annotated as predicted or unknown proteins (Tables 1 and 2).

**Table 1:** Identification of AR-responsive proteins in *L. formosana*.

Spot^a^	NCBI accession^b^	Protein identity^c^	Thero. Da/p*I* ^d^	Exper. Da/pI^e^	SC^f^	MP/TP^g^	Score^h^	C^i^	Organism matched
**Material metabolism**									
L1	gi|297835714	glucose-1-phosphate adenylyltransferase (GPAT)	57.66/6.54	46.40/5.48	15%	6/7	81	D	*Arabidopsis lyrata* subsp
L2	gi|226500818	shikimate kinase family protein	30.34/5.87	27.41/4.74	36%	7/8	110	U	*Zea mays*
L3	gi|162458456	zeta-carotene desaturase	63.49/8.62	39.22/6.95	10%	5/5	74	U	*Zea mays*
L4	gi|356504466	haloalkane dehalogenase-like	45.01/8.43	15.10/4.12	21%	8/10	99	U	*Glycine max*
L6	gi|95117792	glutamate dehydrogenase (GDH)	44.82/6.38	44.21/7.00	23%	8/8	112	U	*Vitis vinifera*
L7	gi|15081239	glycine-rich protein 17 (GRP17)	53.32/10.31	14.09/4.02	21%	5/5	87	U	*Arabidopsis thaliana*
L8	gi|15218536	stearoyl-acyl-carrier protein desaturase-like protein (SACPDLP)	44.36/6.10	17.36/5.09	14%	6/6	97	U	*Arabidopsis thaliana*
L9	gi|38426301	6-phosphogluconate dehydrogenase	51.78/6.58	16.60/4.74	12%	5/5	78	U	*Oryza sativa*
L11	gi|255567778	cysteine synthase (CS)	43.38/7.60	20.47/5.49	21%	9/10	114	D	*Ricinus communis*
L12	gi|3341511	cinnamoyl-CoA reductase	40.63/5.73	47.97/4.40	16%	5/5	81	U	*Saccharum officinarum*
L39	gi|309951612	flavanone 3-hydroxylase (F3H)	41.24/5.39	39.35/5.41	28%	7/9	103	U	*Litchi chinensis*
L46	gi|114795072	chalcone synthase (CHS)	42.83/6.05	45.37/6.91	22%	8/10	84	U	*Pyrus communis*
**Photosynthesis and energy production**									
L18	gi|1022805	phosphoglycerate kinase (PGK)	41.99/4.93	36.02/5.79	24%	8/11	108	D	*Arabidopsis thaliana*
L19	gi|355329944	actin	40.37/5.67	41.03/5.13	44%	15/28	144	U	*Malus domestica*
L20	gi|225423755	photosystem II stability/assembly factor HCF136	44.47/6.92	34.91/5.31	36%	12/27	109	D	*Vitis vinifera*
L21	gi|357438645	chlorophyllide a oxygenase (CAO)	25.99/8.95	90.68/5.69	30%	4/4	76	D	*Medicago truncatula*
L22	gi|183217735	ATP synthase CF1 alpha subunit	55.62/5.20	32.35/4.95	24%	10/11	138	U	*Guizotia abyssinica*
L23	gi|114421	ATP synthase subunit beta	59.93/5.95	59.97/5.09	25%	12/25	88	U	*Nicotiana plumbaginifolia*
L24	gi|225428086	V-type proton ATPase subunit B	54.37/5.04	66.71/4.68	32%	16/40	107	U	*Vitis vinifera*
L25	gi|147945622	oxygen-evolving enhancer protein (OEE)	34.72/6.08	36.12/4.91	24%	7/8	109	U	*Leymus chinensis*
L26	gi|5758863	ATP synthase beta subunit	53.56/5.16	73.58/5.31	42%	17/24	177	U	*Colchicum autumnale*
L27	gi|158726716	ribulose 1,5-bisphosphate carboxylase/oxygenase activase	48.81/6.10	46.81/5.10	26%	10/13	115	D	*Flaveria bidentis*
L28	gi|15222551	phosphoribulokinase (PPK)	44.72/5.71	43.22/5.22	24%	9/18	87	D	*Arabidopsis thaliana*
L29	gi|79322651	fructose-bisphosphate aldolase	41.95/5.94	39.20/5.66	15%	7/7	96	D	*Arabidopsis thaliana*
L30	gi|2108252	P-glycoprotein-2 (PGP2)	135.75/8.97	38.31/6.98	6%	7/7	77	D	*Arabidopsis thaliana*
L31	gi|162946539	ribulose-1,5-bisphosphate carboxylase/oxygenase small subunit	20.80/8.23	28.36/6.01	32%	5/5	97	D	*Solanum tuberosum*
L32	gi|1022805	phosphoglycerate kinase (PGK)	41.99/4.93	42.14/5.61	24%	8/17	87	D	*Arabidopsis thaliana*
L33	gi|356539332	RuBisCO large subunit-binding protein subunit alpha-like isoform 1	61.73/5.23	79.85/4.53	19%	9/11	111	D	*Glycine max*
L34	gi|146188415	ribulose-1,5-biphosphate carboxylase/oxygenase (Rubisco)	46.17/6.44	87.79/4.87	26%	11/21	104	D	*Podalyria canescens*
L61	gi|297816654	metal ion binding protein	26.66/9.55	16.60/4.74	25%	4/4	73	U	*Arabidopsis lyrata* subsp
**Stress and defense**									
L35	gi|42568255	TIR-NBS-LRR class disease resistance protein	121.85/6.36	35.08/5.30	7%	7/7	81	U	*Arabidopsis thaliana*
L36	gi|241989446	NBS-LRR class disease resistance protein	19.52/5.43	24.37/6.42	29%	4/4	75	U	*Oryza sativa*
L37	gi|224111296	cc-nbs-lrr resistance protein	149.57/5.73	24.48/5.95	13%	12/16	106	U	*Populus trichocarpa*
L38	gi|289157416	1-hydroxy-2-methyl-2-(E)-butenyl 4-diphosphate reductase	51.64/5.63	28.42/5.08	16%	5/5	77	U	*Artemisia annua*
L40	gi|14210363	ascorbate peroxidase (APX)	27.50/5.13	29.55/6.55	22%	4/4	73	U	*Zantedeschia aethiopica*
L41	gi|110289462	glutathione S-transferase (GST)	18.04/10.13	37.47/5.70	36%	4/4	74	U	*Oryza sativa*
L43	gi|18404004	TSK-associating protein 1	84.20/4.58	56.88/4.55	10%	6/7	74	U	*Arabidopsis thaliana*
L44	gi|17530547	class III peroxidase ATP32	35.02/6.88	58.60/6.71	30%	6/6	107	U	*Arabidopsis thaliana*
L45	gi|356559803	stromal 70 kDa heat shock-related protein	73.88/5.20	16.24/4.95	19%	12/23	100	U	*Glycine max*
L47	gi|357490825	NBS-LRR resistance protein	136.09/6.05	48.01/5.37	8%	9/9	102	U	*Medicago truncatula*
**Signal transduction**									
L48	gi|333441302	phytochrome C	42.23/6.08	41.46/4.94	21%	6/7	86	U	*Digoniopterys microphylla*
L49	gi|371940268	truncate phytochrome A2 protein	110.22/6.65	48.59/5.56	9%	9/9	103	U	*Glycine max*
L50	gi|18405351	abscisic acid receptor PYL6	24.06/6.17	46.80/6.69	22%	4/4	74	U	*Arabidopsis thaliana*
L51	gi|359475476	serine carboxypeptidase-like 18	58.18/6.16	25.30/5.39	18%	7/7	108	U	*Vitis vinifera*
L62	gi|77553062	cyclic nucleotide-gated ion channel 14	72.47/8.15	23.34/5.66	10%	7/7	91	D	*Oryza sativa*
**Transcription**									
L52	gi|187369233	topoisomerase I	102.34/9.50	22.01/5.39	13%	11/16	88	U	*Catharanthus roseus*
L53	gi|308802618	DNA-damage-inducible protein F	48.73/4.50	34.58/6.99	16%	6/8	80	U	*Ostreococcus tauri*
L54	gi|20196900	putative RNA helicase A	124.80/6.54	34.89/4.69	8%	8/10	74	U	*Arabidopsis thaliana*
L55	gi|126022792	RNA polymerase beta subunit	155.17/9.36	41.14/4.63	10%	10/12	104	U	*Spinacia oleracea*
L56	gi|308808201	minichromosome maintenance protein 10 isoform 1-like	64.87/9.42	65.31/6.50	17%	9/11	95	U	*Ostreococcus tauri*
L57	gi|323690255	maturase K	45.41/9.80	48.01/5.37	16%	5/5	80	U	*Pitraea cuneato-ovata*
L58	gi|183529139	maturase K	60.34/9.28	28.91/4.96	12%	7/8	82	U	*Jacqueshuberia brevipes*
L59	gi|21629786	maturase K	46.79/9.62	134.26/6.81	21%	7/8	100	U	*Hyalochlamys globifera*
L60	gi|255567202	putative transcription elongation factor s-II	38.76/9.57	46.80/5.67	14%	5/5	77	D	*Ricinus communis*
**Post-translational modification**									
L13	gi|224089629	f-box family protein	45.92/6.43	27.54/5.11	16%	5/5	82	U	*Populus trichocarpa*
L14	gi|308802882	ubiquitin-protein ligase/hyperplastic discs protein	92.22/5.72	14.48/5.58	8%	7/7	81	U	*Ostreococcus tauri*
L15	gi|304322967	translational elongation factor Tu (EF-Tu)	45.64/6.16	14.88/5.89	20%	6/6	94	D	*Floydiella terrestris*
L16	gi|225429488	eukaryotic initiation factor 4A-11	47.07/5.38	47.10/5.26	17%	10/10	118	D	*Vitis vinifera*
L17	gi|30684767	cell division protease ftsH-2	74.28/6.00	83.96/5.15	15%	9/9	123	D	*Arabidopsis thaliana*
**Hormone response**									
L5	gi|3024127	S-adenosylmethionine synthase (SAM synthase)	43.43/5.51	48.33/5.91	31%	10/29	82	U	*Catharanthus roseus*
L10	gi|33342178	ABA inducible protein	17.52/5.95	27.92/5.00	24%	5/5	84	U	*Triticum aestivum*
L42	gi|350535769	ethylene-responsive transcriptional coactivator	16.08/10.03	39.11/6.27	36%	6/6	112	U	*Solanum lycopersicum*
**Others**									
L63	gi|168047657	predicted protein	113.56/6.45	58.41/6.85	12%	10/13	83	U	*Physcomitrella patens* subsp
L64	gi|302772723	hypothetical protein	84.02/9.40	24.97/4.47	10%	6/6	79	U	*Selaginella moellendorffii*
L65	gi|302823293	hypothetical protein ELMODRAFT-449095	84.19/9.43	36.83/4.51	15%	8/11	84	U	*Selaginella moellendorffii*
L66	gi|167998464	predicted protein	35.11/9.51	16.16/4.89	22%	7/8	101	U	*Physcomitrella patens* subsp
L67	gi|297797753	predicted protein	41.46/6.47	42.91/5.14	11%	5/5	78	U	*Arabidopsis lyrata* subsp
L68	gi|147834872	hypothetical protein VITISV-040309	46.87/6.06	46.55/5.38	32%	17/33	116	U	*Vitis vinifera*
L69	gi|168004878	predicted protein	46.87/5.63	34.19/5.72	27%	9/22	92	U	*Physcomitrella patens* subsp
L70	gi|168000362	predicted protein	120.48/9.19	47.05/5.29	12%	10/13	86	U	*Physcomitrella patens* subsp
L71	gi|116779860	unknown	23.62/7.77	59.88/6.98	29%	6/7	99	U	*Picea sitchensis*
L72	gi|218201086	hypothetical protein OsI-29089	83.26/6.03	27.24/6.61	16%	8/10	86	D	*Oryza sativa*
L73	gi|49389230	hypothetical protein	38.17/6.67	65.34/5.50	21%	7/7	99	D	*Oryza sativa*
L74	gi|297721931	Os03g0229600	11.76/9.77	29.86/5.71	41%	5/5	107	D	*Oryza sativa*

The seedlings were treated with AR (pH 3.0) for one month. The pH of AR solution was adjusted with a mixture of H_2_SO_4_ and HNO_3_ in the ratio of 5∶1. The final concentration of H_2_SO_4_ and HNO_3_ were 0.45 and 0.09 mM, respectively.

a Spot No. is the unique differentially expressed protein spot number. L, protein spot in *L. formosana* gel.^ b^ Database accession numbers according to NCBInr. ^c^ Description of the proteins identified by MALDI-TOF MS. ^d^ Theoretical mass (kDa) and pI of identified proteins. ^e^ Experimental mass (kDa) and pI of identified proteins. ^f^ Amino acid sequence coverage for the identified proteins. ^g^ Number of the matched peptides and the total searched peptides. ^h^Mascot searched score against the database NCBInr. Protein score is −10*Log(*P*), where *P* is the probability that the observed match is a random event. Protein scores greater than 73 are significant (*p*<0.05).^ i^ Spot abundance change. D decreased abundance of proteins, U increased abundance of protein.

**Table 2:** Identification of AR-responsive proteins in *S. superba*.

Spot^a^	NCBI accession^b^	Protein identity^c^	Thero. Da/p*I* ^d^	Exper. Da/p*I* ^e^	SC^f^	MP/TP^g^	Score^h^	C^i^	Organism matched
**Material metabolism**									
S1	gi|62321345	glutamate-ammonia ligase	23.45/5.70	44.39/5.55	20%	6/8	93	U	*Arabidopsis thaliana*
S2	gi|205277664	granule-bound starch synthase I (GBSS)	15.08/6.41	20.84/5.29	34%	4/4	78	U	*Thinopyrum intermedium*
S3	gi|60101355	glutamine synthetase (GS)	31.14/5.75	43.58/5.76	23%	6/8	91	U	*Vigna radiata*
S19	gi|303280145	glycosyltransferase family 7 protein	48.84/9.23	53.35/5.17	16%	5/5	79	U	*Micromonas pusilla*
**Photosynthesis and energy production**									
S6	gi|255544584	phosphoglycerate kinase (PGK)	50.11/8.74	42.11/5.39	36%	13/25	143	D	*Ricinus communis*
S7	gi|37721507	photosystem II subunit H	2.53/11.72	49.18/4.85	62%	3/4	75	D	*Ixiolirion tataricum*
S8	gi|5708095	ATP synthase gamma chain	33.48/6.12	37.32/5.66	35%	9/20	99	D	*Arabidopsis thaliana*
S9	gi|6688696	ribulose-1,5-bisphosphate carboxylase/oxygenase large subunit	52.03/6.22	20.08/5.97	20%	10/18	95	D	*Morkillia mexicana*
S11	gi|290490212	ATP synthase CF1 alpha subunit protein	55.58/5.04	70.29/5.01	32%	17/27	173	U	*Staphylea colchica*
S12	gi|13430334	rubisco activase	37.25/6.70	51.86/4.85	33%	10/18	99	D	*Zantedeschia aethiopica*
S13	gi|308320553	ribulose-1,5-bisphosphate carboxylase/oxygenase large subunit	30.68/6.24	26.45/5.67	22%	5/6	78	D	*Madia* sp
S14	gi|170664996	ribulose-1,5-bisphosphate carboxylase/oxygenase large subunit	52.85/6.00	53.02/6.05	23%	11/16	125	U	*Lycoseris crocata*
S10	gi|81301612	protein Ycf2	268.41/8.58	61.08/5.64	9%	16/20	99	U	*Nicotiana tomentosiformis*
S20	gi|303283276	beta carbonic anhydrase	26.07/6.21	24.62/5.62	27%	5/5	92	D	*Micromonas pusilla*
**Stress and defense**									
S16	gi|3328221	thioredoxin peroxidase (TPx)	28.40/6.34	19.81/4.56	37%	5/8	86	U	*Secale cereale*
S17	gi|2654208	heat shock 70	76.27/5.19	105.00/4.39	21%	12/22	116	U	*Spinacia oleracea*
S18	gi|116323	endochitinase 3	37.17/8.72	47.33/4.89	30%	5/6	84	U	*Nicotiana tabacum*
S21	gi|384247250	clavaminate synthase-like protein	41.35/5.60	38.59/5.86	17%	5/5	81	U	*Coccomyxa subellipsoidea*
**Signal transduction**									
S15	gi|350536755	14-3-3 protein 4	29.44/4.66	28.05/4.37	43%	6/9	99	U	*Solanum lycopersicum*
S22	gi|115393868	phytocyanin-like arabinogalactan-protein (PLA)	18.87/9.17	77.80/7.03	34%	4/4	77	U	*Gossypium hirsutum*
S27	gi|110532561	calmodulin (CaM)	17.10/4.06	41.79/5.06	39%	4/4	81	U	*Aegiceras corniculatum*
S28	gi|224131906	calcium dependent protein kinase 6 (CDPK)	62.93/5.37	45.18/4.96	13%	7/8	93	U	*Populus trichocarpa*
**Transcription**									
S23	gi|108861639	transposase	15.02/9.08	30.68/4.32	47%	5/7	82	U	*Oligostachyum sulcatum*
S24	gi|379041605	maturase K	40.48/10.01	22.60/4.71	18%	8/11	80	D	*Bromus commutatus*
S25	gi|255660958	pentatricopeptide repeat-containing protein	39.59/5.95	21.25/5.73	21%	8/10	98	U	*Verbena macdougalii*
S26	gi|11993344	marpoflo protein	27.93/9.19	20.25/5.88	19%	5/5	74	D	*Marchantia polymorpha*
**Post-translational modification**									
S4	gi|225441985	proteasome subunit alpha type-5 isoform 1	26.13/4.65	25.55/4.35	31%	6/8	84	U	*Vitis vinifera*
S5	gi|356545337	mitochondrial import receptor subunit TOM6 homolog isoform 1	6.27/9.36	21.22/6.80	62%	4/5	79	U	*Glycine max*
**Others**									
S29	gi|224064392	predicted protein	37.25/5.62	33.24/4.68	37%	7/10	102	U	*Populus trichocarpa*
S30	gi|388496926	unknown	39.81/8.46	44.78/5.11	24%	8/10	120	D	*Lotus japonicus*
S31	gi|242052501	hypothetical protein SORBIDRAFT_03g010120	48.42/9.53	27.93/5.71	22%	9/12	106	D	*Sorghum bicolor*
S32	gi|168071263	predicted protein	23.61/9.95	81.80/4.52	26%	4/4	80	U	*Physcomitrella patens* subsp
S33	gi|77554095	hypothetical protein LOC_Os12g13240	23.16/10.25	27.91/6.30	34%	6/9	92	U	*Oryza sativa*
S34	gi|145347277	predicted protein	107.30/5.81	35.29/5.92	9%	7/7	78	U	*Ostreococcus lucimarinus*

The seedlings were treated with AR (pH 3.0) for one month. The pH of AR solution was adjusted with a mixture of H_2_SO_4_ and HNO_3_ in the ratio of 5∶1. The final concentration of H_2_SO_4_ and HNO_3_ were 0.45 and 0.09 mM, respectively.

a Spot No. is the unique differentially expressed protein spot number. S, protein spot in *S. superba* gel.^ b^ Database accession numbers according to NCBInr. ^c^ Description of the proteins identified by MALDI-TOF MS. ^d^ Theoretical mass (kDa) and pI of identified proteins. ^e^ Experimental mass (kDa) and pI of identified proteins. ^f^ Amino acid sequence coverage for the identified proteins. ^g^ Number of the matched peptides and the total searched peptides. ^h^Mascot searched score against the database NCBInr. Protein score is −10*Log(*P*), where *P* is the probability that the observed match is a random event. Protein scores greater than 73 are significant (*p*<0.05).^ i^ Spot abundance change. D decreased abundance of proteins, U increased abundance of proteins.

Of the 74 protein spots identified in *L. formosana*, the abundances of 53 proteins were increased and those of 21 proteins were decreased in response to AR (Table 1). In *S. superba*, 21 proteins were increased in their abundances and 11 proteins were decreased under AR stress (Table 2). Among these affected proteins, phosphoglycerate kinase (PGK, spot L32, L8, S6) and ATP synthase CF1 alpha subunit (spot L22, S11), were decreased in their abundances in both *L. formosana* and *S. superba* (Tables 1 and 2). Remarkably, after AR treatment, abundance of maturase K was increased in *L. formosana*, but decreased in *S. superba* (Tables 1 and 2). Further analysis on the results revealed that some proteins were represented by more than one spot. These proteins included phosphoglycerate kinase (PGK, spot L32, L8), ATP synthase beta subunits (spot L22, L26) and maturase K (spot L57–59) in *L. formosana* and ribulose-1,5-bisphosphate carboxylase/oxygenase (Rubisco) large subunit (spot S9, S13, S14) in *S. superba.* The multiple spots might represent isoforms or different post-translation modification of individual proteins [13].

### Functional classification of AR-responsive proteins

**Figure 4:**
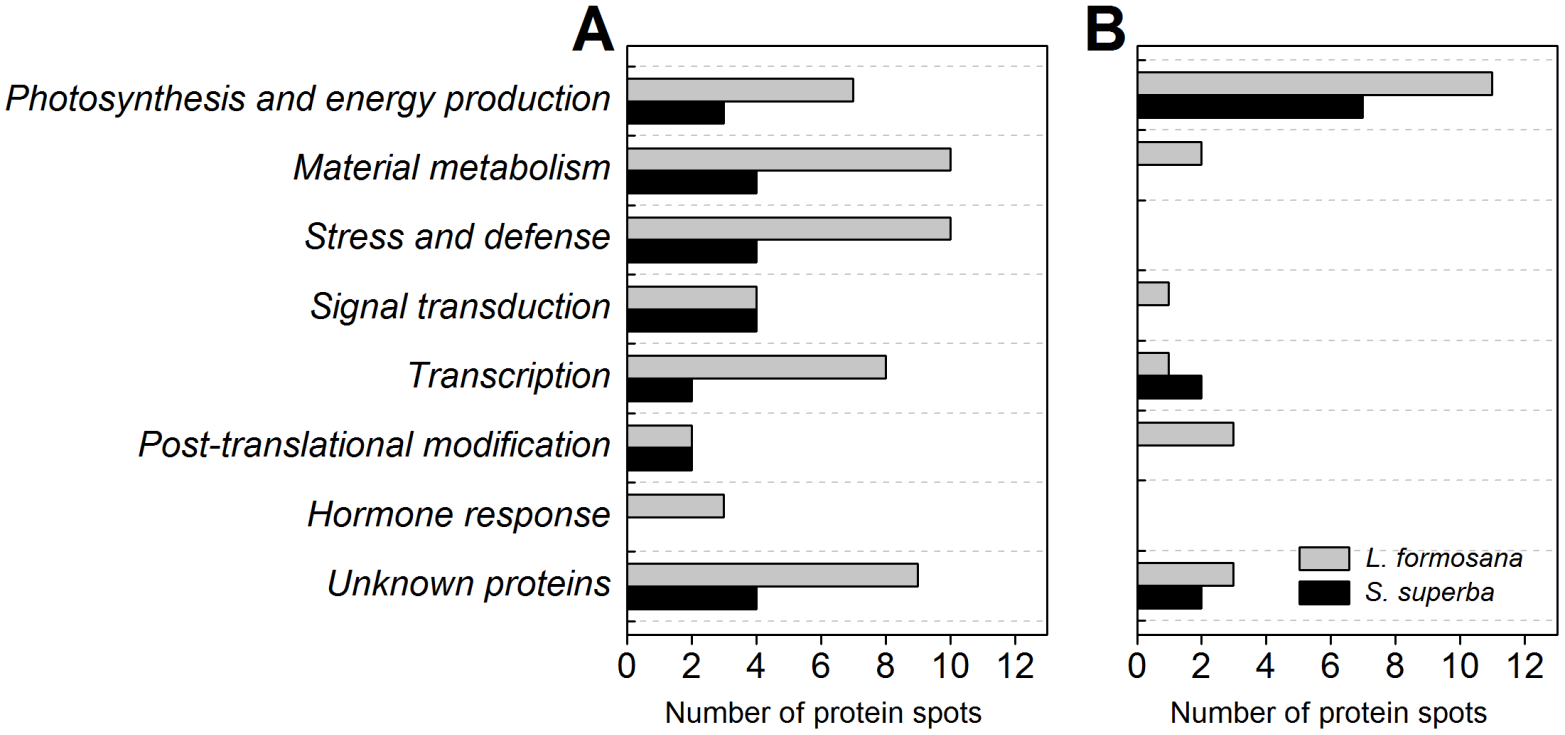
Number of protein spots significantly changed in AR-treated *L. formosana* and *S. superba*. (A) Protein spots increased in their abundances. (B) Protein spots decreased in their abundances.

**Figure 5:**
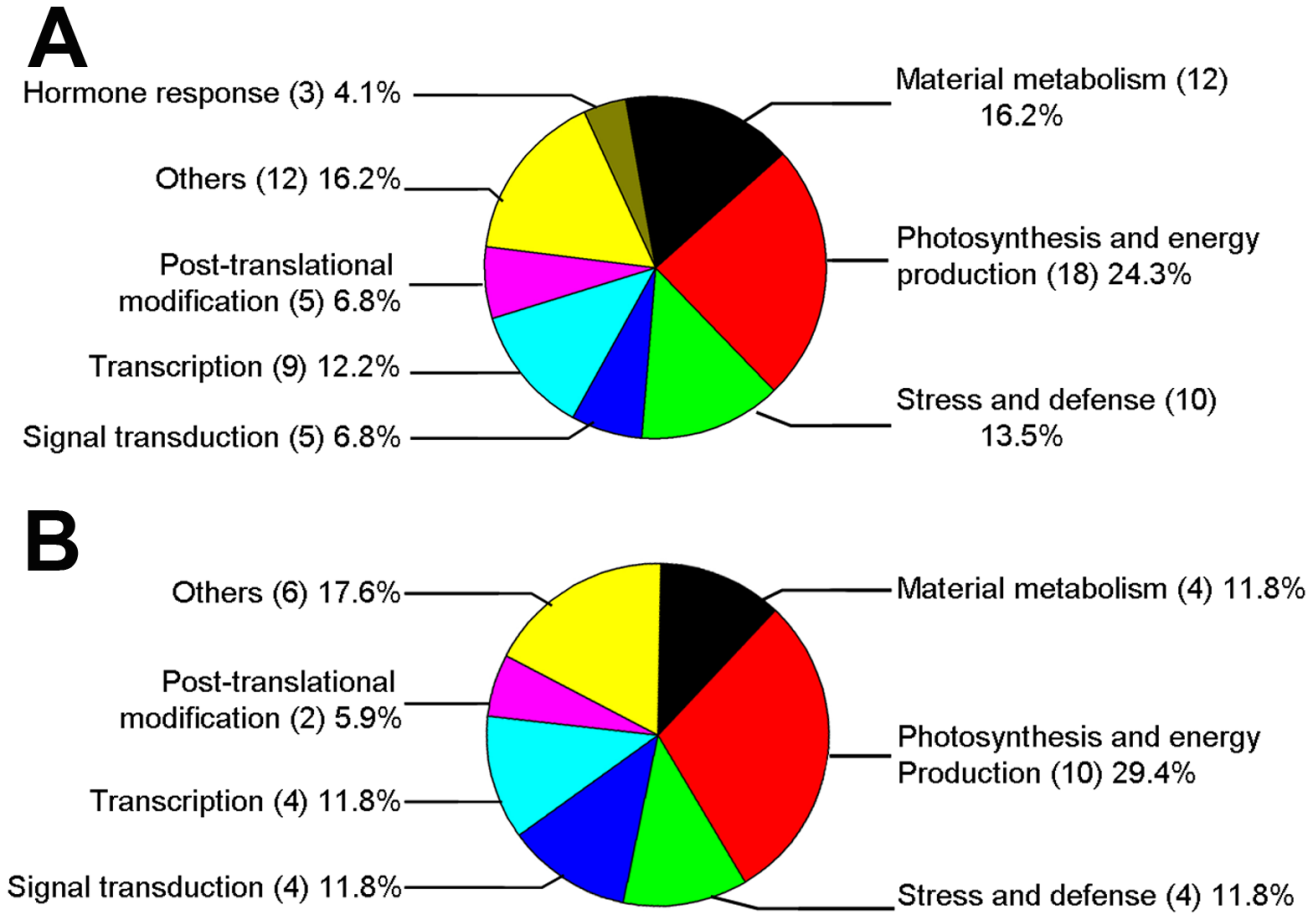
Functional classification of AR-responsive proteins in *L. formosana* (A) and *S. superba* (B). The proportion of identities in each functional group was the sum of this identity accounting for all protein quantities.

In order to obtain annotation of AR-responsive protein, all identified proteins were further classified according to their biological function and cellular component categories in UniProt (http://www.uniprot.org) and/or NCBI protein database (http://www.ncbi.nlm.nih.gov). AR-responsive proteins were found to be involved in a wide range of biological processes. After AR treatment, with the exception of photosynthesis and energy production related proteins, the abundance of most proteins were decreased in both *L. formosana* and *S. superba* (Fig. 4). As shown in Fig. 5, a higher percentage of proteins were involved in photosynthesis and energy production, which accounted for 24.3% and 29.4% of the total proteins in *L. formosana* and *S. superba*, respectively. The following groups were proteins involved in material metabolism, stress and defense, transcription, post-translational and modification, and signal transduction. In opposition to *L. formosana*, no protein related to hormone response was found in *S. superba*.

### Protein expression analysis by Western blot

**Figure 6:**
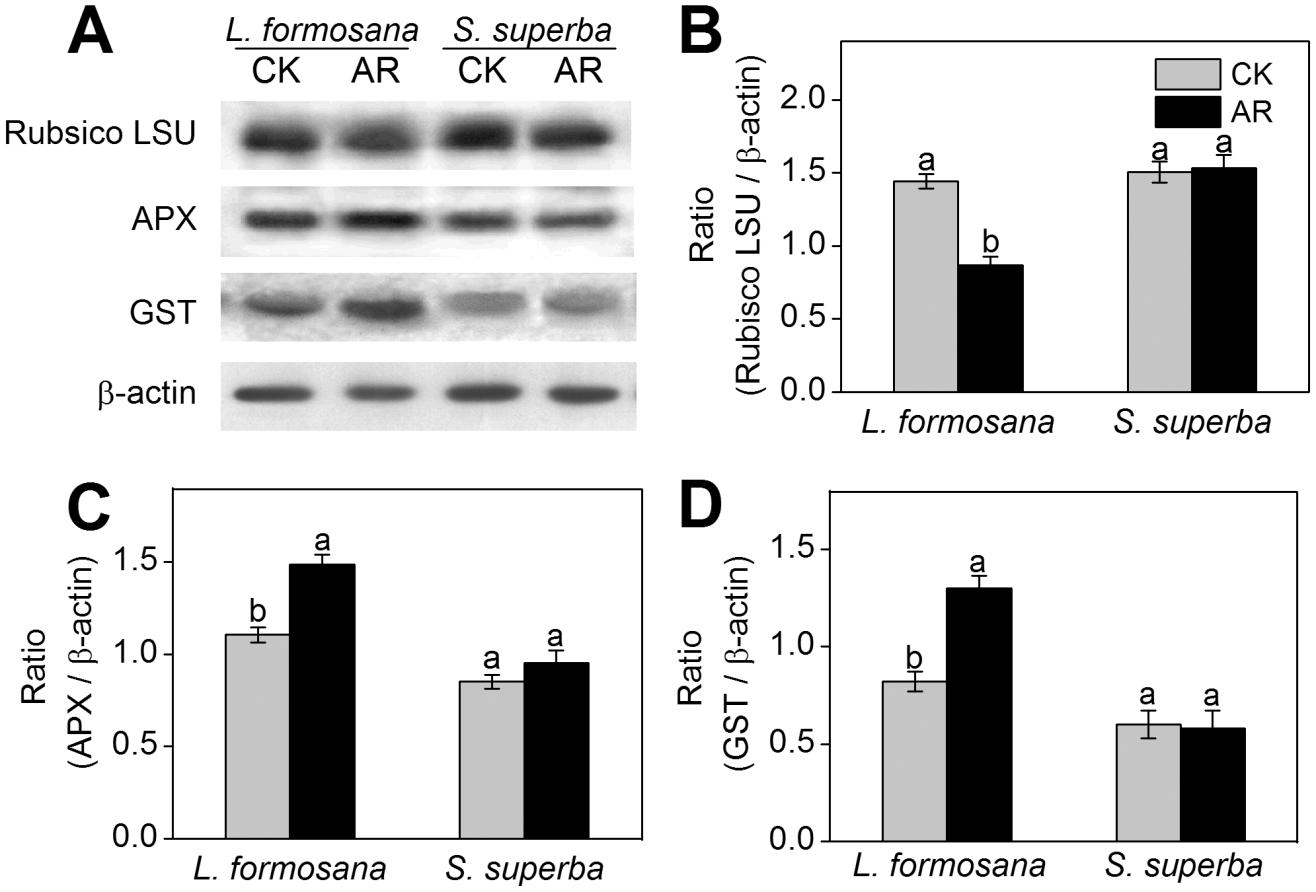
Western blot analysis showing the expression of three protein spots. (A) Expression of rubulose-1,5-bisphoshate carboxylase large subunit (RuBisco LSU), ascorbate peroxidase (APX) and glutathione-S-transferase (GST) in *L. formosana* and *S. superba* seedlings after AR treatment. Relative expression level of RuBisco LSU (B), APX (C) and GST (D) were analyzed with the Quantity One software. *β*-actin was used as the internal control. Means with different letters indicate significantly difference (*p*<0.05) with regard to AR treatments.

Our above proteomic results revealed that the abundance of Rubisco was decreased (spot L31, L34), while APX (spot L40) and GST (spot L41) were increased in *L. formosana* under AR treatment (Table 1). As shown in Fig. 6A and B, compared with the control, the protein expression of Rubisco large subunit analyzed by Western blot was significantly decreased in AR-treated *L. formosana.* In contrast, the protein expression level of APX and GST increased 1.3-fold and 1.6-fold, respectively, compared to control, in *L. formosana* (Fig. 6C and D). No significant change in the expression of Rubisco large subunit, APX and GST was observed in *S. superba* (Fig. 6).

## Discussion

AR has negative effects on plant growth and development [1], [6]. Neves et al [23] found that simulated AR (pH 3.1) caused chlorosis and necrosis in leaves and led to chlorophyll loss and photosynthetic depression in *Eugenia uniflora*. Moreover, chlorophyll content and photosynthesis in *L. formosana* were also remarkably suppressed by AR treatment in this study (Fig. 1). Compared with *S. superba*, reductions on chlorophyll content and photosynthetic ability by AR were more obvious in *L. formosana* (Fig. 1), which is consistent with the results of previous studies [9], [11]. Besides chlorophyll content and photosynthesis, proline content, MDA content and ROS (e.g. H_2_O_2_ and O_2_^•-^) production are commonly used as biochemical markers to monitor the damage level in plants under environmental stress [24]. In this study, AR increased proline content, MDA content and ROS production in both *L. formosana* and *S. superba* (Fig. 2), which were consistent with the results obtained by Chen et al [11]. However, the increase in these physiological changes caused by AR was less pronounced in *S. superba* than those in *L. formosana* (Fig. 1 and 2), suggesting that *S. superba*, a tolerant species, had less cell damage than *L. formosana*, a sensitive species.

To further reveal the different strategies to cope with AR between the two species, 74 protein spots in *L. formosana* and 34 protein spots in *S. superba* caused by AR were identified by proteomic analysis in this study. Interestingly, similar results were also reported in previous studies, which reported more changes in protein abundance in sensitive species, *Arabidopsis thaliana*, than in tolerant species, *Thellungiella halophila*, under salt stress [25]. Since *L. formosana* had much higher changes in its protein profile, our results proved that this species is more sensitive to AR than *S. superba*.

### Photosynthesis and energy production-related proteins

Photosynthesis is an essential metabolic process of plants and is vulnerable to environmental stress. It is well known that AR can remarkably reduce photosynthesis [3], [23]. In this study, two light reaction-related proteins, including chlorophyllide a oxygenase (CAO, spot L21) and photosystem II (PSII) stability/assembly factor HCF136 (spot L20), were identified in *L. formosana*. CAO, that converts chlorophyll a to chlorophyll b, regulates the stabilization of light-harvesting chlorophyll a/b proteins [26]. Villeth et al [27] reported that pathogen infection could decrease the accumulation of PSII stability/assembly factor HCF136, which is important for the accurate assembly of PSII. In this study, AR remarkably decreased the abundance of CAO and PSII stability/assembly factor HCF136 in *L. formosana*, but no light reaction-related proteins was depressed by AR in *S. superba* (Table 1 and 2). These results suggest that photosynthesis apparatus of *L. formosana* is more sensitive to AR stress than *S. superba*.

It has been reported that the expression of Calvin cycle enzymes were down-regulated in *Arabidopsis* under salinity stress [25]. Our previous study also reported that the reduction in photosynthesis was linked to Calvin cycle enzymes in AR-treated *Arabidopsis* [16]. Consistent with previous results, our proteomic data from this study confirmed that the abundances of Calvin cycle-related proteins including phosphoribulokinase (PPK, spot L28), Rubisco (spot L31, L34) and Rubisco activase (spot L27) were significantly decreased in *L. formosana* (Table 1). Rubisco, the CO_2_ fixing enzyme in Calvin cycle, is the primary limiting factor of net photosynthesis under environmental stress [16]. Rubisco activase promotes and maintains the catalytic activity of Rubisco [28]. The decreased expression of Rubisco and Rubisco activase in AR-treated *L. formosana* may disturb Calvin cycle activity, leading to the reduction in photosynthetic CO_2_ assimilation and thus the inhibition in plant growth. Moreover, the results of Western blot analysis showed that the decrease in protein expression of Rubisco large subunit was more obvious in *L. formosana* than in *S. superba* under AR stress (Fig. 6A and B). These results may explain why AR-induced damage to photosynthesis was more serious in *S. superba* than in *L. formosana* (Fig. 1D and E). Compared with *L. formosana*, less damage of AR to photosynthesis-related proteins probably result from higher tolerance to AR in *S. superba.*

Besides photosynthesis-related proteins, AR also affected the abundances of energy production-related proteins in *L. formosana* and *S. superba.* It is well known that increased ATP production is required in response to abiotic stress in plants [29]. For example, the abundance of ATP synthase was considerably increased in salt-stressed rice [30] and osmotic-stressed wheat [31]. Thus, the increased abundances of ATP synthase subunits in *L. formosana* (spot L22, L23, L26) and *S. superba* (spot S11) in our study demonstrate the prime role of ATP synthase in the adaptation of two tree species to AR stress.

### Material metabolism-related proteins

In *L. formosana*, AR affected a series of protein abundances involved in several metabolism processes, including nitrogen metabolism (spot L2, L6, L11), starch and sugar metabolism (spot L1, L9), lipid metabolism (spot L7, L8), secondary metabolism (spot L39, L46), vitamin metabolism (spot L3) and lignin biosynthesis (spot L12) (Table 1). However, only two nitrogen metabolism-related proteins (spot S1, S3) and one starch biosynthesis-related protein (spot S2) were remarkably induced by AR in *S. superba* (Table 2). It is clear that AR disturbed more metabolism processes in *L. formosana* than in *S. superba*.

AR stress can change free amino acid levels and disturbs N metabolism in plants [19]. Cysteine synthase (CS, spot L11), which is responsible for the terminal step of cysteine biosynthesis, is a critical enzyme involved in environmental stress response in plants [12]. The abundance of CS was decreased in *L. formosana* (Table 1), indicating that metabolic processes related to cysteine biosynthesis might be strongly depressed by AR. In addition, two glutamine metabolism-related proteins, such as glutamate dehydrogenase (GDH, spot L6) and glutamine synthetase (GS, spot S3), were identified in *L. formosana* and *S. superba*, respectively. Abiotic stresses, such as salinity, drought and metal toxicity, can up-regulate *GDH* gene expression and enhance GDH activity in plants [32]. GS catalyzes ATP-dependent incorporation of ammonium into glutamate and other reduced N compounds [33]. It has been found that GS accumulation was also stimulated by salt and drought, and this helped improve the tolerance of plants to stresses [25]. In agreement with previous findings, our study found that the abundance of GDH in *L. formosana*, as well as that of GS in *S. superba*, were increased (Table 1), suggesting that GDH and GS play critical roles in N metabolic acclimation of plants when exposed to AR.

Abiotic stress can also affect starch synthesis [34]. In this study, two starch biosynthesis-related proteins including glucose-1-phosphate adenylyltransferase (GPAT, spot L1) in *L. formosana* and granule-bound starch synthase (GBSS, spot S2) in *S. superba* were identified (Tables 1 and 2). Interestingly, the responses of GPAT and GBSS to AR were different in two broad-leaf species. The abundance of GPAT was decreased in *L. formosana*. However, GBSS, the only enzyme implicated in amylose synthesis [35], was increased in AR-treated *S. superba*. Basically, this result was consistent with the observation where GBSS activity and starch content in rice was found to increase under cold stress [36]. It is well known that starch is required to synthesize sucrose which serves as a carbon and energy source for plant growth and stress response [35]. Thus we believe that the increased abundance of GBSS may contribute to higher AR-tolerance in *S. superba* through enhancing starch synthesis and energy production. It also should be noted that AR stress changed the abundances of lipid metabolism-related proteins including glycine-rich protein 17 (GRP17, spot L7) and stearoyl-acyl-carrier protein desaturase-like protein (SACPDLP, spot L8), as well as secondary metabolism-related proteins including flavanone 3-hydroxylase (F3H, spot L39) and chalcone synthase (CHS, L46) in *L. formosana*. The abundance of SACPDLP was increased, which may contribute to lipid synthesis and membrane integrity in AR-treated *L. formosana* [25]. F3H and CHS are two key enzymes that catalyze the biosynthesis of flavonoids and chalcones, both of which play critical roles in enhancing secondary metabolism under environmental stress [37]. The increased abundances of these proteins imply that secondary metabolism pathway may be activated to cope with AR stress in AR-sensitive species, *L. formosan*a.

### Stress defense-related proteins

The majority of environmental stresses generate a secondary oxidative stress in plants [13]. Oxidative stress occurs when there is a serious imbalance between ROS production and antioxidant defense [24]. Overproduction of ROS induced by a number of adverse environmental factors can attack proteins, lipids, and nucleic acids [38]. Under long-term heavy metal or AR stress, significant accumulation of ROS was observed in previous studies [7], [19], [39]. For instance, Kovacik et al [38] observed the increased ROS in four *Tillandsia* species under 2 µM Cd^2+^ treatment over 30 days. In this study, H_2_O_2_ and O_2_^•-^ content, and thus oxidative stress, induced by AR was remarkably higher in *L. formosana* than in *S. superba* (Fig. 2C and D).

To avoid oxidative damage, plants developed an antioxidant system consisting of antioxidative enzymes as well as non-enzymatic antioxidants [38]. In *L. formosana*, three antioxidant-related proteins, including APX (spot L40), GST (spot L41) and class III peroxidase ATP32 (spot L44), were identified by proteomic analysis. APX plays an important role in scavenging H_2_O_2_ from cells [34]. GST is also an important enzyme that counteracts cellular damage induced by oxidative stress [12]. Enhanced activity of APX and GST has also regularly been detected in plants after exposure to salt, cold, heavy metal, and heat stresses [40]. Our recently published work found that both APX and GST were increased in their abundance in an AR-sensitive conifer tree species, *Piuns massoniana* [41]. Similarity, APX (spot L40) and GST (spot L41) with higher abundances and expression levels (Table 1 and Fig. 6) were also found in AR-treated *L. formosana* in this study, implying that antioxidant defense system was provoked by AR in *L. formosana*. However, the expression levels of APX and GST were not changed in *S. superba* seedlings under AR treatment (Fig. 6). A likely reason is that there may be other pathways that can remove excessive ROS in *S. superba*. Interestingly, thioredoxin peroxidase (TPx, spot S16), which appears to be a key enzyme in H_2_O_2_ detoxification [42], was found to have an increased abundance in AR-treated *S. superba*. The increased TPx may function in resisting AR-induced oxidative damage by reducing ROS production in *S. superba*. In addition, endochitinase, a glycosyl hydrolase that catalyzes chitin degradation, plays an essential role in forming the fine cell-wall matrix that enhances the physical barrier against abiotic stresses [43]. Tapia et al [44] reported that endochitinase could be induced by heat, drought, and salinity. In this study, the increased abundance of endochitinase (spot S18) in *S. superba* (Table 2) may improve physical interactions at the plasma membrane-cell wall interface to cope with AR stress.

### Signal transduction-related proteins

Signal transduction plays a crucial role in triggering a cascade of defense events [12]. Phytochrome is a chromoprotein that regulates the expressions of a large number of light-responsive genes and controls plant growth and development [45]. Recently, phytochrome has been found to modulate both biotic and abiotic stresses, such as salinity, drought, cold or herbivory [46]. Cross-talk between phytochrome-mediated light signals and some stress signaling pathways has been reported in diverse plants [47]. Thus it is possible that phytochrome is involved in the modulation of AR stress. A recent study found that increased abundance of phytochrome was needed to resist cold stress in cucumber [48]. Likewise, AR also increased the abundance of both phytochrome C (spot L48) and truncate phytochrome A2 protein (spot L49) in *L. formosana* but not in *S. superba* in this study (Table 1), which suggest a role of phytochrome signaling in response to AR in this sensitive species. In addition to the phytochrome signaling pathway, antioxidant enzymes have been found to be modulated by phytochromes under stress conditions [46]. In this study, the increased expression of antioxidant enzymes (APX and GST) was observed in *L. formosana* under AR treatment (Fig. 6). We suggest that the enhancement of phytochromes induced by AR modulates the antioxidant system in *L. formosana* seedlings. Further research need to widen our understanding of the role of phytochrome in AR-stressed plants.

Calcium (Ca) also plays a crucial role in regulating plant defense responses to various environmental stimuli [49]. Recently, Kovacik et al [50] found that oxidative stress evoked by hexavalent chromium were evidently suppressed by Ca in *Matricaria chamomilla* using microscopic visualization method. Our previous study also reported that Ca addition dramatically alleviated the negative effects of AR on seed germination, seedling growth and photosynthesis [3]. Free Ca ion within cell is a second messenger for conveying internal and external signals by Ca sensors that subsequently regulate diverse cellular processes in plants [17]. Calmodulin (CaM) and calcium-dependent protein kinase (CDPK), two major types of Ca sensor, play important roles in Ca signaling and further response to diverse stresses in plants [51]. Saijo et al [52] found that over-expression of *OsCDPK* gene enhanced tolerance to cold, salt and drought in transgenic rice. Moreover, 14-3-3 protein can also regulate proteins involved in stress response and activate CDPK signal transduction pathway in plants [13]. In *S. superba*, the abundances of CaM (spot S27), CDPK (spot S28) and 14-3-3 protein (spot S15) were increased after AR treatment, while these proteins were not identified in *L. formosana*. In agreement with the proteomic analysis results, the enhanced gene expression of *CaM* and *CDPK* by real-time quantitative PCR was more obvious in *S. superba* than in *L. formosana* under AR treatment (Fig. S1). These results indicate that AR activated different signaling transduction pathways in two tree species and that Ca sensor-dependent signaling pathway might play a critical role in enhancing AR tolerance in *S. superba*.

### AR affected transcription, protein synthesis and modification

Transcription machinery plays an important role in abiotic stress adaptation [25]. Nine proteins (spots L52–L60) and four proteins (spots S23–S26) that involve in gene transcription were identified in response to AR stress in *L. formosana* and *S. superba*, respectively (Tables 1 and 2). Four of these proteins were maturase K (spots L57–59, S24). In plants, maturase K catalyzes intron RNA binding during reverse transcription and spicing and directly affects gene expression at transcriptional level [53]. The changes in protein expression of maturase K are very complex in plants under abotic stress, depending on plant species and the type of stress [54]. Pandey et al [54] reported that maturase K was induced when plant suffered from high ROS pressure. On the contrary, maturase K protein was down-regulated in salt-treated maize [55]. In this study, the abundance of maturase K (spots L57–59) was increased in *L. formosana*, but was decreased in *S. superba* (Tables 1 and 2). Based on our physiological data, AR induced higher ROS (H_2_O_2_ and O_2_^•-^) production in *L. formosana* than that in *S. superba* (Fig. 2), which might be one reason to explain the increased abundance of maturase K in *L. formosana.* However, the decreased abundance of maturase K in *S. superba* indicates that its gene transcription had been affected by AR, thought no significant visible damage symptom emerged in *S. superba* leaves.

Translational elongation factor Tu (EF-Tu) plays important roles in response to abiotic stresses including high and low temperatures, salinity, and water deficit [56]. Pandey et al [54] reported that the expression of EF-Tu was down-regulated in chickpea with the increased treatment period of water deficit, which was consistent with the decrease in EF-Tu abundance (spot L15) after AR stress in *L. formosana* observed in our study. This result indicates that AR has induced a lower protein synthesis in *L. formosana*. However, EF-Tu was not identified in *S. superba*, instead, proteasome subunit alpha type-5 isoform 1 (spot S4) and mitochondrial import receptor subunit TOM6 homolog isoform 1 (spot S5) were found in AR-treated leaves (Table 2). Proteasome degrades the proteins with translational errors and proteins damaged by stress which can aggregate and become toxic to the cell [57]. Proteasome has been implicated in regulating numerous plant signaling and metabolic pathways under stress condition [57]. Our pervious study found that the expression of 20 S proteasome subunit was up-regulated in *Arabidopsis* after 32 h of AR treatment [16]. In this study, the abundance of proteasome subunit alpha type-5 isoform 1 was also increased in AR-treated *S. superba*, indicating that the control of protein degradation by the proteasome is likely to play an important role in enhancing AR-tolerance in *S. superba*. In addition, mitochondrial import receptor subunit TOM complex mediates the translocation and uptake of nuclear-encoded mitochondrial preproteins from the cytosol [58]. Increased abundance of mitochondrial import receptor subunit TOM6 by AR may accelerate the import of mitochondrial preproteins and enhance cellular metabolism and energy production in mitochondria, finally contributing to improve resistant to AR in *S. superba*.

### Hormone response-related proteins

Plant hormones are not only important in plant growth and development, but also closely related to environmental stresses response [59]. It is known that increased ethylene biosynthesis is a general response of plants to stress conditions [60]. S-adenosyl-L-methionine (SAM), a major methyl donor in plants, is used as a substrate for ethylene biosynthesis [60]. SAM synthetase catalyzes SAM biosynthesis from L-methionine and ATP [61]. It has been found that both activity and expression of SAM synthetase were increased in tomato and rice under salt stress [62]. Accordingly, our present results indicate that AR increased the abundance of SAM synthetase (spot L5) in *L. formosana* (Table 1), which may further contribute to ethylene biosynthesis. Another ethylene-related protein, ethylene-responsive transcriptional coactivator (spot L42), which was also increased by AR stress in *L. formosana*, can positively control the expression of ethylene-responsive genes in plants [63]. In addition, ABA inducible protein (spot L10), which is involved in ABA stimulus and abiotic stresses response, was increased in *L. formosana* when exposed to AR. It appeared that ethylene biosynthesis and ABA signaling pathways might be activated in AR-treated *L. formosana*. It is interesting to note that, in *S. superba*, no hormone response-related proteins was identified, partly due to the high resistance to AR stress in *S. superba*.

## Conclusion

Using the approach of proteomic analysis, this study investigated the differential responses to AR in two broad-leaf tree species, *L. formosana* and *S. superba*, an AR-sensitive species and an AR-tolerant species, respectively. After AR treatment, more proteins were significantly changed in their abundances in *L. formosana* than in *S. superba*. It should be noted that hormone response-related protein was only found in *L. formosana*. After AR treatment, signaling pathways, energy production and antioxidant system were activated in both *L. formosana* and *S*. superba. Due to higher AR-tolerance in *S. superba*, AR induced less damage to photosynthesis-related proteins in this species. Moreover, the proteins related to starch synthesis and translation were depressed in AR-treated *L. formosana*, but enhanced in *S. superba*, implying that these proteins may greatly contribute to enhance AR-tolerance in *S. superba.* The identification of novel AR-responsive proteins in this study provides not only new insights into AR stress responses, but also a good starting point for further exploration of the differential AR adaptation strategies between sensitive and tolerant tree species.

## Supporting Information

Figure S1 Effects of AR on *CaM* and *CDPK* gene expressions in *L. formosana* and *S. superba.* The relative gene expression level was calculated based on AR/CK.
(TIF)

Table S1 Details of identified proteins and peptides list of each protein in AR-treated *L. formosana*.
(DOC)

Table S2 Details of identified proteins and peptides list of each protein in AR-treated *S. superba.*
(DOC)
