# Supplementary material for: Proteomic Analysis Reveals Differences in Tolerance to Acid Rain in Two Broad-Leaf Tree Species, Liquidambar formosana and Schima superba
Source: PLoS One. 2014 Jul 15;9(7):e102532. doi: 10.1371/journal.pone.0102532 (PMC4099204; doi:10.1371/journal.pone.0102532)
Supplement: Table S1 — Details of identified proteins and peptides list of each protein in AR-treated L. formosana. (DOC) [file pone.0102532.s002.doc]

**Supplemental Table S1. Details of identified proteins and peptides list of each protein in AR-treated *L. formosana*.**

| **Spot^a^** | **NCBI  accession^b^** | **Protein identity^c^** | **Peptide sequence** |
| --- | --- | --- | --- |
| **Material metabolism** | | | |
| L1 | gi|297835714 | glucose-1-phosphate adenylyltransferase (GPAT) | R.KPLSFSSSSLTGEKLSTPEK.T |
|  |  |  | R.SVLGIILGGGAGTR.L |
|  |  |  | R.AKPAVPLGANYR.L |
|  |  |  | K.IYVLTQFNSASLNR.H |
|  |  |  | K.VDTTILGLDDER.A |
|  |  |  | K.KPVPDFSFYDR.S |
|  |  |  |  |
| L2 | gi|226500818 | shikimate kinase family protein | R.AATAAATGFFSPSTVPPR.R |
|  |  |  | R.RFSSVTPPASLCTAR.C |
|  |  |  | R.AFPSLEIPLEELNPSVDLLRR.T |
|  |  |  | R.NIAKLLANSIIYR.Y |
|  |  |  | K.LLANSIIYR.Y |
|  |  |  | R.HGVSIWIDVPLEMAANDMLK.S |
|  |  |  | R.AKEMMEAAGKPF.- + Oxidation (M) |
|  |  |  |  |
| L3 | gi|162458456 | zeta-carotene desaturase | K.GSPDVYLSGPIKK.Y |
|  |  |  | R.EAPGNDPFRPDQK.T |
|  |  |  | K.TPVKNFFLSGSYTK.Q |
|  |  |  | R.RTSAYICGAGEELLALR.K |
|  |  |  | R.TSAYICGAGEELLALR.K |
|  |  |  |  |
| L4 | gi|356504466 | haloalkane dehalogenase-like | -.MTLQAFHGSACCFR.S |
|  |  |  | -.MTLQAFHGSACCFR.S + Oxidation (M) |
|  |  |  | R.SHLLHFSSSTASSPPFLASRR.F |
|  |  |  | R.RFQSCPSTR.K |
|  |  |  | R.FQSCPSTRK.S |
|  |  |  | K.AKDPIFGLAMGSGSQATGDR.F |
|  |  |  | R.RPYLTSGSSGFALNAISRNMNK.D |
|  |  |  | R.NMNK.D |
|  |  |  |  |
| L6 | gi|95117792 | glutamate dehydrogenase (GDH) | K.SLLIPFR.E |
|  |  |  | K.VECTIPKDDGTLASFVGFR.V |
|  |  |  | K.DDGTLASFVGFR.V |
|  |  |  | K.GGIGCNPGELSLSELER.L |
|  |  |  | K.FHGYSPAVVTGKPTDLGGSLGR.D |
|  |  |  | K.FIIEAANHPTDPEADEILSK.K |
|  |  |  | R.MGAFTLGVNR.V |
|  |  |  | R.MGAFTLGVNR.V + Oxidation (M) |
|  |  |  |  |
| L7 | gi|15081239 | glycine-rich protein 17 (GRP17) | K.SEGEEGMSSGDEGMSGSEGGMSGGEGGK.S + Oxidation (M) |
|  |  |  | K.SKSGK.G |
|  |  |  | K.GMSGGSESEEGMSGSEGGMSGGGGSKSK.S + 3 Oxidation (M) |
|  |  |  | K.GMSGGMSGSEEGMFGSEGGMSSGGGSK.S + Oxidation (M) |
|  |  |  | K.GMSGGMSGSEEGMSGSEGGMSGGGGGKSK.S + 4 Oxidation (M) |
|  |  |  |  |
| L8 | gi|15218536 | stearoyl-acyl-carrier protein desaturase-like protein (SACPDLP) | K.HQNKIHTMPPEK.M + Oxidation (M) |
|  |  |  | R.AWTAEENR.H |
|  |  |  | R.TYLYLSGR.V |
|  |  |  | R.TYLYLSGRVDMLMVER.T + Oxidation (M) |
|  |  |  | K.LLEIDPNGAVSAVADMMR.K + 2 Oxidation (M) |
|  |  |  | K.LLEIDPNGAVSAVADMMRK.K + 2 Oxidation (M) |
|  |  |  |  |
| L9 | gi|38426301 | 6-phosphogluconate dehydrogenase | -.MGQKLALNIAEK.G + Oxidation (M) |
|  |  |  | R.AEAEGALPVLGHR.D |
|  |  |  | R.VAAAGVLKAEGMPSGLLETINVDK.K + Oxidation (M) |
|  |  |  | K.AEGMPSGLLETINVDKK.M + Oxidation (M) |
|  |  |  | K.ICSYAQGMNLLR.A + Oxidation (M) |
|  |  |  |  |
| L11 | gi|255567778 | cysteine synthase (CS) | K.TPMVYLNNIVK.G |
|  |  |  | K.LEIMEPCCSVK.D |
|  |  |  | K.LILTMPASMSMER.R |
|  |  |  | K.LILTMPASMSMER.R + Oxidation (M) |
|  |  |  | K.AFGAELVLTDSAK.G |
|  |  |  | K.EKNPNIK.V |
|  |  |  | K.IQGIGAGFVPR.N |
|  |  |  | K.LIAVVFPSFGER.Y |
|  |  |  | R.YLSSVLFQSIR.E |
|  |  |  |  |
| L12 | gi|3341511 | cinnamoyl-CoA reductase | K.GTVRNPDDPK.N |
|  |  |  | R.NWYCYGKAVAEQAAWDAAR.Q |
|  |  |  | R.DVADAHLRVFESPR.A |
|  |  |  | R.VLHR.E |
|  |  |  | K.GHLPVLGEQTTEADDK.E |
|  |  |  |  |
| L39 | gi|309951612 | flavanone 3-hydroxylase (F3H) | M.ALATLTALAQEKTLNASFVR.D |
|  |  |  | R.EFFALPPEEK.L |
|  |  |  | R.EFFALPPEEKLR.F |
|  |  |  | K.GGFIVSSHLQGEAVQDWR.E |
|  |  |  | K.CPQSDLTLGLKR.H |
|  |  |  | R.HTDPGTITLLLQDQVGGLQATR.D |
|  |  |  | R.EGEKPILEQPITFAEMYR.R + Oxidation (M) |
|  |  |  |  |
| L46 | gi|114795072 | chalcone synthase (CHS) | K.ENPSVCEYMAPSIDAR.Q |
|  |  |  | K.ENPSVCEYMAPSIDAR.Q + Oxidation (M) |
|  |  |  | K.LLGLRPSVK.R |
|  |  |  | R.LMMYQQGCFAGGTVLR.L |
|  |  |  | R.LMMYQQGCFAGGTVLR.L + Oxidation (M) |
|  |  |  | R.VLVVCSEITAVTFR.G |
|  |  |  | R.EVGLTFHLLK.D |
|  |  |  | R.QVLSDYGNMSSACVLFILDEVR.R + Oxidation (M) |
| **Photosynthesis and energy production** | | | |
| L18 | gi|1022805 | phosphoglycerate kinase (PGK) | R.ADLNVPLDDNQTITDDTR.I |
|  |  |  | K.FSLAPLVPR.L |
|  |  |  | K.KLASLADLYVNDAFGTAHR.A |
|  |  |  | K.LASLADLYVNDAFGTAHR.A |
|  |  |  | K.FLKPSVAGFLLQK.E |
|  |  |  | K.ELDYLVGAVSNPK.R |
|  |  |  | K.RPFAAIVGGSK.V |
|  |  |  | K.GVSLLLPTDVVVADK.F |
|  |  |  |  |
| L19 | gi|355329944 | actin | K.AGFAGDDAPR.A |
|  |  |  | R.AVFPSIVGRPR.H |
|  |  |  | R.HTGVMVGMGQK.D + 2 Oxidation (M) |
|  |  |  | K.YPIEHGIVSNWDDMEK.I |
|  |  |  | K.YPIEHGIVSNWDDMEK.I + Oxidation (M) |
|  |  |  | K.IWHHTFYNELR.V |
|  |  |  | R.VAPEEHPVLLTEAPLNPK.A |
|  |  |  | R.LDLAGRDLTDSLMK.I |
|  |  |  | K.LAYVALDYEQELETAK.S |
|  |  |  | K.NYELPDGQVITIGAER.F |
|  |  |  | R.KDLYGNIVLSGGSTMFPGIADR.M |
|  |  |  | K.DLYGNIVLSGGSTMFPGIADR.M |
|  |  |  | K.DLYGNIVLSGGSTMFPGIADR.M + Oxidation (M) |
|  |  |  | K.DLYGNIVLSGGSTMFPGIADRMSR.E + Oxidation (M) |
|  |  |  | K.GEYDESGPSIVHR.K |
|  |  |  |  |
| L20 | gi|225423755 | photosystem II stability/assembly factor HCF136 | K.DGGNTWVPR.S |
|  |  |  | R.SIPSAEDEDFNYR.F |
|  |  |  | K.SAEMVTDEGAIYVTSNR.G |
|  |  |  | K.SAEMVTDEGAIYVTSNR.G + Oxidation (M) |
|  |  |  | R.AAVQETVSATLNR.T |
|  |  |  | R.TVSSGISGASYYTGTFNTVNR.S |
|  |  |  | R.GNFYLTWEPGQPYWQPHNR.A |
|  |  |  | R.RIQNMGWR.A |
|  |  |  | R.ADGGLWLLVR.G |
|  |  |  | R.GFGILDVGYR.S |
|  |  |  | R.DKAADNIAANLYSVK.F |
|  |  |  | K.GFVLGNDGVLLR.F |
|  |  |  |  |
| L21 | gi|357438645 | chlorophyllide a oxygenase (CAO) | K.GKFGVFAVFGEADK.K |
|  |  |  | R.HDIEYLDWRAR.Q |
|  |  |  | K.LAYMEELVNDRLLQDR.S + Oxidation (M) |
|  |  |  | K.SLNVSGPVQSYHPNLKNFWYPVAFSADLK.D |
|  |  |  |  |
| L22 | gi|183217735 | ATP synthase CF1 alpha subunit | M.VTIQADEISNIIR.E |
|  |  |  | R.ERIEQYNR.E |
|  |  |  | K.IVNTGTVLQVGDGIAR.I |
|  |  |  | R.IAQIPVSEAYLGR.V |
|  |  |  | R.VINALAKPIDGR.G |
|  |  |  | R.LIESPAPGIISR.R |
|  |  |  | R.SVYEPLQTGLIAIDSMIPIGR.G + Oxidation (M) |
|  |  |  | K.ASSVAQVVTTFQER.G |
|  |  |  | R.HTLIIYDDLSK.Q |
|  |  |  | R.TYLK.T |
|  |  |  |  |
| L23 | gi|114421 | ATP synthase subunit beta | K.VCQVIGAVVDVR.F |
|  |  |  | R.LVLEVAQHLGENMVR.T |
|  |  |  | R.LVLEVAQHLGENMVR.T + Oxidation (M) |
|  |  |  | R.VLNTGSPITVPVGR.A |
|  |  |  | K.VVDLLAPYQR.G |
|  |  |  | K.AHGGFSVFAGVGER.T |
|  |  |  | K.CALVYGQMNEPPGAR.A |
|  |  |  | K.CALVYGQMNEPPGAR.A + Oxidation (M) |
|  |  |  | R.VGLTGLTVAEHFR.D |
|  |  |  | R.DAEGQDVLLFIDNIFR.F |
|  |  |  | R.FTQANSEVSALLGR.I |
|  |  |  | R.QISELGIYPAVDPLDSTSR.M |
|  |  |  |  |
| L24 | gi|225428086 | V-type proton ATPase subunit B | K.YQEIVNIR.L |
|  |  |  | K.TPVSLDMLGR.I |
|  |  |  | K.TPVSLDMLGR.I + Oxidation (M) |
|  |  |  | R.TYPEEMIQTGISTIDVMNSIAR.G + Oxidation (M) |
|  |  |  | R.TYPEEMIQTGISTIDVMNSIAR.G + 2 Oxidation (M) |
|  |  |  | K.IPLFSAAGLPHNEIAAQICR.Q |
|  |  |  | K.RDFEENGSMER.V + Oxidation (M) |
|  |  |  | R.DFEENGSMER.V + Oxidation (M) |
|  |  |  | R.EEVPGRR.G |
|  |  |  | R.GYPGYMYTDLATIYER.A |
|  |  |  | R.GYPGYMYTDLATIYER.A + Oxidation (M) |
|  |  |  | R.QIYPPINVLPSLSR.L |
|  |  |  | R.DHADVSNQLYANYAIGK.D |
|  |  |  | K.AVVGEEALSSEDLLYLEFLDKFER.K |
|  |  |  | R.KFVAQGAYDTR.N |
|  |  |  | K.FVAQGAYDTR.N |
|  |  |  |  |
| L25 | gi|147945622 | oxygen-evolving enhancer protein (OEE) | K.RLTFDEIQSK.T |
|  |  |  | K.AGKYEMK.K + Oxidation (M) |
|  |  |  | K.KFCLEPTSFTVK.A |
|  |  |  | K.FCLEPTSFTVK.A |
|  |  |  | K.NEPPAFQK.T |
|  |  |  | R.LTYTLDEMEGPLEVGADGTLK.F + Oxidation (M) |
|  |  |  | K.FEEKDGIDYAAVTVQLPGGER.V |
|  |  |  |  |
| L26 | gi|5758863 | ATP synthase beta subunit | R.GRIAQIIGPVLDVVFSPGR.M |
|  |  |  | R.MPNIYNALVVK.G + Oxidation (M) |
|  |  |  | R.GMEVIDTGAPLSVPVGGATLGR.I |
|  |  |  | R.IFNVLGEPVDNLGPVDTR.T |
|  |  |  | K.LSIFETGIK.V |
|  |  |  | K.VVDLLAPYR.R |
|  |  |  | K.AHGGVSVFGGVGER.T |
|  |  |  | R.EGNDLYMEMK.E + 2 Oxidation (M) |
|  |  |  | K.VALVYGQMNEPPGAR.M |
|  |  |  | K.VALVYGQMNEPPGAR.M + Oxidation (M) |
|  |  |  | R.VGLTALTMAEYFR.D |
|  |  |  | R.VGLTALTMAEYFR.D + Oxidation (M) |
|  |  |  | R.DVNEQDVLLFIDNIFR.F |
|  |  |  | R.FVQAGSEVSALLGR.M |
|  |  |  | K.GIYPAVDPLDSTSTMLQPR.I |
|  |  |  | R.IVGEEHYETAQR.V |
|  |  |  | K.AMNLEEESKL.- + Oxidation (M) |
|  |  |  |  |
| L27 | gi|158726716 | ribulose 1,5-bisphosphate carboxylase/oxygenase activase | R.GLAYDMSDDQQDITR.G |
|  |  |  | K.SFQCELVFAK.M |
|  |  |  | K.MGITPIMMSAGELESGNAGEPAK.L |
|  |  |  | R.VPIIVTGNDFSTLYAPLIR.D |
|  |  |  | K.FYWAPTR.D |
|  |  |  | R.IGVCIGIFR.T |
|  |  |  | K.LVDTFPGQSIDFFGALR.A |
|  |  |  | R.VYDDEVR.K |
|  |  |  | R.VYDDEVRK.W |
|  |  |  | R.EGPPTFEQPK.M |
|  |  |  |  |
| L28 | gi|15222551 | phosphoribulokinase (PPK) | R.ANDFDLMYEQVK.A |
|  |  |  | K.ILVIEGLHPMFDER.V |
|  |  |  | R.VRDLLDFSIYLDISNEVK.F |
|  |  |  | R.DLLDFSIYLDISNEVK.F |
|  |  |  | R.DMAERGHSLESIK.A + Oxidation (M) |
|  |  |  | R.KPDFDAFIDPQK.Q |
|  |  |  | R.LDELIYVESHLSNLSTK.F |
|  |  |  | K.FYGEVTQQMLK.H |
|  |  |  | K.FYGEVTQQMLK.H + Oxidation (M) |
|  |  |  |  |
| L29 | gi|79322651 | fructose-bisphosphate aldolase | K.RLASIGLENTEANR.Q |
|  |  |  | R.LASIGLENTEANR.Q |
|  |  |  | K.MVDVLVEQNIVPGIK.V |
|  |  |  | K.MVDVLVEQNIVPGIK.V + Oxidation (M) |
|  |  |  | K.EAAWGLAR.Y |
|  |  |  | R.ATPEQVASYTLKLLR.N |
|  |  |  | K.EGMFVKGYTY.- |
|  |  |  |  |
| L30 | gi|2108252 | P-glycoprotein-2 (PGP2) | K.LINIIGLAYLFPK.Q |
|  |  |  | R.EALENTYK.Y |
|  |  |  | R.EALENTYKYGR.K |
|  |  |  | K.LGKVDGHIQFK.D |
|  |  |  | R.VMVGRTTVVVAHR.L + Oxidation (M) |
|  |  |  | K.GNQMVASVFEILDRK.T + Oxidation (M) |
|  |  |  | K.VGERGVQMSGGQR.Q |
|  |  |  |  |
| L31 | gi|162946539 | ribulose-1,5-bisphosphate carboxylase/oxygenase small subunit | -.MASSVISSAAVATR.T + Oxidation (M) |
|  |  |  | M.ASSVISSAAVATR.T |
|  |  |  | R.VRCMQVWPPINTK.K + Oxidation (M) |
|  |  |  | K.YETLSYLPDLTDEQLLK.E |
|  |  |  | K.SPGYYDGRYWTMWK.L + Oxidation (M) |
|  |  |  |  |
| L32 | gi|1022805 | phosphoglycerate kinase (PGK) | R.ADLNVPLDDNQTITDDTR.I |
|  |  |  | K.FSLAPLVPR.L |
|  |  |  | K.KLASLADLYVNDAFGTAHR.A |
|  |  |  | K.LASLADLYVNDAFGTAHR.A |
|  |  |  | K.FLKPSVAGFLLQK.E |
|  |  |  | K.ELDYLVGAVSNPK.R |
|  |  |  | K.RPFAAIVGGSK.V |
|  |  |  | K.GVSLLLPTDVVVADK.F |
|  |  |  |  |
| L33 | gi|356539332 | RuBisCO large subunit-binding protein subunit alpha-like isoform 1 | K.LADAVGLTLGPR.G |
|  |  |  | R.NVVLDEFGSPK.V |
|  |  |  | K.LGLLSVTSGANPVSLK.R |
|  |  |  | K.TVQGLVEELEKK.A |
|  |  |  | R.GYISPQFVTNPEK.L |
|  |  |  | K.APGFGER.R |
|  |  |  | K.DSTTIIADAATKDELQAR.V |
|  |  |  | K.VGAATETELEDR.K |
|  |  |  | K.APVAGAPQGLTV.- |
|  |  |  |  |
| L34 | gi|146188415 | ribulose-1,5-biphosphate carboxylase/oxygenase (Rubisco) | -.MSPPTETK.A + Oxidation (M) |
|  |  |  | K.DTDILAAFR.V |
|  |  |  | K.TFQGPPHGIQVER.D |
|  |  |  | R.GGLDFTKDDENVNSQPFMR.W |
|  |  |  | R.FLFCAEALYK.A |
|  |  |  | K.GHYLNATAGTCEEMIK.R |
|  |  |  | R.DNGLLLHIHR.A |
|  |  |  | R.QKNHGMHFR.V + Oxidation (M) |
|  |  |  | K.NHGMHFR.V |
|  |  |  | K.NHGMHFR.V + Oxidation (M) |
|  |  |  | R.EITLGFVDLLRDDFVEK.D |
|  |  |  |  |
| L61 | gi|297816654 | metal ion binding protein | -.MKANMFYCASQASTATANDER.T + 2 Oxidation (M) |
|  |  |  | R.SFTAPCSSGDDYVAPYR.Q |
|  |  |  | R.SNSGSLMKLISSDVSLAR.K |
|  |  |  | R.GCQGKVK.K |
| **Stress and defense** | | | |
| L35 | gi|42568255 | TIR-NBS-LRR class disease resistance protein | K.IVTDVSDKLNLTPSR.D |
|  |  |  | R.KLDVVNGFNILADR.S |
|  |  |  | K.LDVVNGFNILADRSLVR.I |
|  |  |  | R.DVLTKGTGTESVK.G |
|  |  |  | K.SIDMSFSYSLK.E + Oxidation (M) |
|  |  |  | R.SITIPLSPGTLSASSR.F |
|  |  |  | R.SITIPLSPGTLSASSRFK.A |
|  |  |  |  |
| L36 | gi|241989446 | NBS-LRR class disease resistance protein | K.LPQEIQKLK.Q |
|  |  |  | R.STGIEELPWEIGELK.Q |
|  |  |  | R.NTRISELPSQIGELK.H |
|  |  |  | R.LPEGVCEDLIK.G |
|  |  |  |  |
| L37 | gi|224111296 | cc-nbs-lrr resistance protein | R.QIGYVLNCNTNIQNLK.N |
|  |  |  | R.QIGYVLNCNTNIQNLKNEVEK.L |
|  |  |  | K.EVLAVVSHTPDIR.R |
|  |  |  | K.HSVRLVAAEVAR.R |
|  |  |  | K.ASCLLLEGDKDGSVK.M |
|  |  |  | K.MHDVVHSFAISVALR.D |
|  |  |  | K.DPSLQIPDSFFREMK.E |
|  |  |  | K.LKVLSLMSSNIVR.L + Oxidation (M) |
|  |  |  | K.VTRLQLLDLSNCER.L |
|  |  |  | K.WETEGSSSQRNNACLSELK.H |
|  |  |  | K.ICHGQLMAESLGNLRILK.V |
|  |  |  | K.SMEEIVVPEDIGEGKMMSK.M + Oxidation (M) |
|  |  |  |  |
| L38 | gi|289157416 | 1-hydroxy-2-methyl-2-(E)-butenyl 4-diphosphate reductase | M.ASLQLTPLSTRTDYLSLPADIK.V |
|  |  |  | K.ENNYEYTWGNVTVK.L |
|  |  |  | K.EAFMEKFK.S + Oxidation (M) |
|  |  |  | K.AGIANQTTMLKGETEEIGK.L |
|  |  |  | K.VVEDALLKVFEIK.R |
|  |  |  |  |
| L40 | gi|14210363 | ascorbate peroxidase (APX) | K.NCAPLMLR.L |
|  |  |  | R.LAWHSAGTFDVCTKSGGPFGTMR.L |
|  |  |  | K.GSDHLR.Q |
|  |  |  | K.YAADEDAFFADYSEAHLK.L |
|  |  |  |  |
| L41 | gi|110289462 | glutathione S-transferase (GST) | -.MAHHHFFAPRK.Q + Oxidation (M) |
|  |  |  | K.RELDGMDQEVWADMGTAVR.V + Oxidation (M) |
|  |  |  | K.QIAKMSSTNSSGDPAAVR.V + Oxidation (M) |
|  |  |  | R.VVGGWASPFMNR.V |
|  |  |  |  |
| L43 | gi|18404004 | TSK-associating protein 1 | K.IQSENQNNTTVTDK.N |
|  |  |  | K.MLEEIEHEFEAASDSLK.Q + Oxidation (M) |
|  |  |  | R.EFEAATESLKQLQVDDSTEDK.E |
|  |  |  | K.QLQVDDSTEDK.E |
|  |  |  | R.KSMLEEIER.E + Oxidation (M) |
|  |  |  | K.EVATEKQTAVDTHFATAK.K |
|  |  |  |  |
| L44 | gi|17530547 | class III peroxidase ATP32 | -.MVRANIVSMVLLMHAIVGFPFHAR.G + 3 Oxidation (M) |
|  |  |  | R.MLFHDCFIEGCDASILLDSTK.D |
|  |  |  | K.EKIENR.C |
|  |  |  | K.IENRCPGVVSCADIVAMAAR.D |
|  |  |  | R.DAVFWAGGPYYDIPK.G |
|  |  |  | R.NDFDNAYFNALQMK.S |
|  |  |  |  |
| L45 | gi|356559803 | stromal 70 kDa heat shock-related protein | R.QAVVNPENTFFSVK.R |
|  |  |  | R.QAVVNPENTFFSVKR.F |
|  |  |  | R.KMSEVDEESK.Q |
|  |  |  | K.QFAAEEISAQVLR.K |
|  |  |  | K.AVVTVPAYFNDSQR.T |
|  |  |  | R.IAGLEVLR.I |
|  |  |  | R.IINEPTAASLAYGFEK.K |
|  |  |  | K.QALQRLTETAEK.A |
|  |  |  | R.AKFEELCSDLLDR.L |
|  |  |  | K.FEELCSDLLDR.L |
|  |  |  | K.SEVFSTAADGQTSVEINVLQGER.E |
|  |  |  | K.NQADSVVYQTEK.Q |
|  |  |  |  |
| L47 | gi|357490825 | NBS-LRR resistance protein | R.EDDK.E |
|  |  |  | R.DSDFLSVYPIVGLGGVGK.T |
|  |  |  | R.LSYFHLTPTLK.R |
|  |  |  | R.LSYFHLTPTLKR.C |
|  |  |  | K.RCFAFCAMFPK.D |
|  |  |  | R.TLFQLNHYTKTK.H |
|  |  |  | K.TKHDYSPTNR.S |
|  |  |  | K.GNSLAELHDLNLGGK.L |
|  |  |  | K.GLNDVCSLSEAQAANLMGK.K + Oxidation (M) |
| **Signal transduction** | | | |
| L48 | gi|333441302 | phytochrome C | K.AISRLQSLPSGNISLLCDVLVK.E |
|  |  |  | K.EVRDLTGYDR.V |
|  |  |  | R.DLTGYDRVMVYK.F + Oxidation (M) |
|  |  |  | K.FHEDEHGEVVSECR.R |
|  |  |  | R.DAPVAIITQSPNAMDLVK.C |
|  |  |  | K.CDGAALYFKNK.T |
|  |  |  |  |
| L49 | gi|371940268 | truncate phytochrome A2 protein | M.STSRPSQSSSNSGR.S |
|  |  |  | R.LQSLPSGSMER.L + Oxidation (M) |
|  |  |  | K.HVRVLQDEK.L |
|  |  |  | R.LWGLVVCHNTTPR.F |
|  |  |  | K.NILR.T |
|  |  |  | R.VTAKDVVFWFR.S |
|  |  |  | K.NVQFEIK.T |
|  |  |  | K.MLLGELFGTHMAACR.L |
|  |  |  | R.LSEQTALKR.L |
|  |  |  |  |
| L50 | gi|18405351 | abscisic acid receptor PYL6 | K.HFVKSCHVVIGDGR.E |
|  |  |  | R.VVSGLPAAFSLER.L |
|  |  |  | R.LMNYK.S + Oxidation (M) |
|  |  |  | R.TRVVESYVVDVPAGNDK.E |
|  |  |  |  |
| L51 | gi|359475476 | serine carboxypeptidase-like 18 | -.MDGQDAILDIQWLKNFSVR.Y + Oxidation (M) |
|  |  |  | K.VASIIFLDSPVGSGFSYAQSSEGYR.T |
|  |  |  | R.TSDSLAAAHGYDFLKK.W |
|  |  |  | R.MTFLSDKLYK.K + Oxidation (M) |
|  |  |  | K.CMEKINLPHVLEPK.C |
|  |  |  | K.FLFSYIWANDR.R |
|  |  |  | K.FLFSYIWANDRR.V |
|  |  |  |  |
| L62 | gi|77553062 | cyclic nucleotide-gated ion channel 14 | K.FRTAFVAPSSR.V |
|  |  |  | K.CYMSYLDCK.T + Oxidation (M) |
|  |  |  | R.DIEEWMRHR.Q |
|  |  |  | R.REIQR.H |
|  |  |  | R.SVTEVEAFALRAEDLK.Y |
|  |  |  | R.YYSHQWR.S |
|  |  |  | K.ARMEDVSSIK.F + Oxidation (M) |
| **Transcription** | | | |
| L52 | gi|187369233 | topoisomerase I | -.MAVEACPTPNLR.D |
|  |  |  | R.DDMDEDDEPIVFK.R + Oxidation (M) |
|  |  |  | R.DDMDEDDEPIVFKR.N |
|  |  |  | K.SPLSSPKVSTSSAK.K |
|  |  |  | K.VKPFNQHK.S |
|  |  |  | K.AYDSDDDKPLAK.K |
|  |  |  | K.VGNFRVEPPGLFR.G |
|  |  |  | K.RIRPCDITINIGK.D |
|  |  |  | K.ELMPGLTAKVFR.T + Oxidation (M) |
|  |  |  | K.EDLKTVALGTSK.I |
|  |  |  | R.HEVPIEKIFNK.S |
|  |  |  |  |
| L53 | gi|308802618 | DNA-damage-inducible protein F | K.ISANGGRASEEGR.R |
|  |  |  | R.ASEEGRR.A |
|  |  |  | R.FCGAHHEPLMMSSGDVMAYADAPTKK.G + 3 Oxidation (M) |
|  |  |  | K.KGILEYGEDYLR.I |
|  |  |  | K.LMMDEGILDFQSVFR.L + Oxidation (M) |
|  |  |  | R.TILLQAVLVR.A |
|  |  |  |  |
| L54 | gi|20196900 | putative RNA helicase A | K.RETCK.I |
|  |  |  | K.IVCTQPRR.I |
|  |  |  | R.RISAMSVSER.I |
|  |  |  | R.TLYLEDVLSILK.S |
|  |  |  | K.ICGDSEDGAILVFLPGWDDINKTR.Q |
|  |  |  | R.QRLLENPFFADSAK.F |
|  |  |  | R.GDGGMHIR.N |
|  |  |  | R.WLPFRTTALEVAQMYILR.E + Oxidation (M) |
|  |  |  |  |
| L55 | gi|126022792 | RNA polymerase beta subunit | R.QSIEIWYSTSEYLRQEMNPNFR.M + Oxidation (M) |
|  |  |  | R.GNVSQVHQLVGMR.G + Oxidation (M) |
|  |  |  | R.DCGTIRGISVSPQNSTMPER.I + Oxidation (M) |
|  |  |  | R.TFHTGGVFTGGTAEHVR.A |
|  |  |  | K.YGTIEMHSIVK.K |
|  |  |  | R.GSFVEVRTNGMIQDFLK.V |
|  |  |  | K.EGSDHTNMNPFYSIYIYPK.T + Oxidation (M) |
|  |  |  | R.QITSKVLVSEDGMSNVFLPGELIGLFR.A |
|  |  |  | K.VLVSEDGMSNVFLPGELIGLFR.A + Oxidation (M) |
|  |  |  | K.VLVSEDGMSNVFLPGELIGLFRAER.T |
|  |  |  |  |
| L56 | gi|308808201 | minichromosome maintenance protein 10 isoform 1-like | R.IGNDGRAYGTWR.V |
|  |  |  | R.NANDDFKASLQK.H |
|  |  |  | K.QHASSIAQAR.K |
|  |  |  | K.QHASSIAQARK.D |
|  |  |  | R.RMELER.E |
|  |  |  | R.GEKPGSDRVVMGDMSNR.N + 2 Oxidation (M) |
|  |  |  | K.APVLKSR.Y |
|  |  |  | R.YDDDAREEDVDTMMTR.M + 2 Oxidation (M) |
|  |  |  | R.FFDSSTCAGHRSCVTQIESIMR.Y |
|  |  |  |  |
| L57 | gi|323690255 | maturase K | -.MEEIQRYLQLER.S |
|  |  |  | R.MYQPNHLIISPNDSTQNR.F + Oxidation (M) |
|  |  |  | R.LVNVFVKVK.D |
|  |  |  | K.DFRASLCLVK.E |
|  |  |  | R.SQILENAFLINNAIKR.L |
|  |  |  |  |
| L58 | gi|183529139 | maturase K | -.MEEFQVYLELDR.S |
|  |  |  | R.LITRMYQQNHLIISANDSNK.N |
|  |  |  | K.DASSFHLLR.L |
|  |  |  | R.SQMLENSFIIENFMK.K + Oxidation (M) |
|  |  |  | R.SQMLENSFIIENFMK.K + 2 Oxidation (M) |
|  |  |  | R.SQMLENSFIIENFMKK.L + 2 Oxidation (M) |
|  |  |  | R.VFLK.R |
|  |  |  |  |
| L59 | gi|21629786 | maturase K | R.LISSLERK.G |
|  |  |  | K.DASSLHFLR.L |
|  |  |  | R.LFLHECHNWDSLITSNSKK.A |
|  |  |  | K.IEHLAEVFAR.A |
|  |  |  | K.DPFMHYVRYQGK.S |
|  |  |  | K.LYNHSIDFLGYR.S |
|  |  |  | K.FDALVPIIPLIRSLAK.S |
|  |  |  |  |
| L60 | gi|255567202 | putative transcription elongation factor s-II | K.QLHRYEDYVSSIQK.Q |
|  |  |  | K.NPDFRR.K |
|  |  |  | R.KILVGEIK.A |
|  |  |  | R.SADEPMTTYVTCTICDNHWK.F + Oxidation (M) |
|  |  |  | R.SADEPMTTYVTCTICDNHWKFC.- |
| **Post-translational modification** | | | |
| L13 | gi|224089629 | f-box family protein | R.IAQFGGGGK.R |
|  |  |  | R.IAQFGGGGKR.S |
|  |  |  | R.GCLCFLANFLGERVDVWMMK.E + Oxidation (M) |
|  |  |  | K.SGDEVLIEHDNLDLCWYDLKR.K |
|  |  |  | R.HLDGRTQDDDEDSK.D |
|  |  |  |  |
| L14 | gi|308802882 | ubiquitin-protein ligase/hyperplastic discs protein | -.MSSEEASAEETPR.K + Oxidation (M) |
|  |  |  | K.SLTEMLLK.V + Oxidation (M) |
|  |  |  | R.NGVIFCSK.D |
|  |  |  | K.VFLK.R |
|  |  |  | K.HVDDLAGNHDQMK.W |
|  |  |  | R.MSAEDRSK.L + Oxidation (M) |
|  |  |  | R.LMTALRHGSTGFAFD.- + Oxidation (M) |
|  |  |  |  |
| L15 | gi|304322967 | translational elongation factor Tu (EF-Tu) | R.KKPHVNIGTIGHVDHGK.T |
|  |  |  | K.IYELMETVDK.H |
|  |  |  | R.EMDKPFLLAVEDVFSITGRGTVATGR.V |
|  |  |  | R.GTVATGRVER.G |
|  |  |  | K.FEGQVYVLTPEEGGRK.T |
|  |  |  | R.EGGRTVGAGMVNR.I |
|  |  |  |  |
| L16 | gi|225429488 | eukaryotic initiation factor 4A-11 | R.GIYAYGFEKPSAIQQR.G |
|  |  |  | R.EDQRILSSGVHVVVGTPGR.V |
|  |  |  | R.ILSSGVHVVVGTPGR.V |
|  |  |  | R.VFDMLR.R |
|  |  |  | R.VFDMLR.R + Oxidation (M) |
|  |  |  | R.RQSLRPDYIK.M |
|  |  |  | R.QSLRPDYIK.M |
|  |  |  | K.MFVLDEADEMLSR.G |
|  |  |  | K.MFVLDEADEMLSR.G + Oxidation (M) |
|  |  |  | R.MLFDIQR.F |
|  |  |  |  |
| L17 | gi|30684767 | cell division protease ftsH-2 | R.FLEYLDKDR.V |
|  |  |  | R.VQLPGLSQELLQK.L |
|  |  |  | K.AKENAPCIVFVDEIDAVGR.Q |
|  |  |  | K.ENAPCIVFVDEIDAVGR.Q |
|  |  |  | R.ADILDSALLRPGR.F |
|  |  |  | R.TPGFSGADLANLLNEAAILAGR.R |
|  |  |  | R.TSISSKEIDDSIDR.I |
|  |  |  | K.QQLFAR.I |
|  |  |  | R.AILSEFTEIPPENR.V |
|  |  |  |  |
| L5 | gi|3024127 | S-adenosylmethionine synthase (SAM synthase) | K.TNMVMVFGEITTKAQVDYEK.I + Oxidation (M) |
|  |  |  | K.AQVDYEK.I |
|  |  |  | K.VLVNIEQQSPDIAQGVHGHLTK.R |
|  |  |  | R.VHTVLISTQHDETVTNDEIAADLK.E |
|  |  |  | K.TIFHLNPSGR.F |
|  |  |  | R.FVIGGPHGDAGLTGR.K |
|  |  |  | K.EILKIVK.E |
|  |  |  | K.ENFDFRPGMIAINLDLK.R |
|  |  |  | K.ENFDFRPGMIAINLDLK.R + Oxidation (M) |
|  |  |  | K.TAAYGHFGR.E |
|  |  |  |  |
| L10 | gi|33342178 | ABA inducible protein | R.TEEK.T |
|  |  |  | K.TGQMMDK.A + 2 Oxidation (M) |
|  |  |  | K.TGQMMDKAGQATEATK.Q |
|  |  |  | K.TAQTAQAAKDR.A |
|  |  |  | K.DQTGSFLGEK.T |
|  |  |  |  |
| L42 | gi|350535769 | ethylene-responsive transcriptional coactivator | -.MPMRPTGGLK.Q + 2 Oxidation (M) |
|  |  |  | -.MPMRPTGGLKQDWDPIVLQKPK.M + 2 Oxidation (M) |
|  |  |  | M.PMRPTGGLKQDWDPIVLQKPK.M + Oxidation (M) |
|  |  |  | K.AATLAVNVRK.L |
|  |  |  | K.LPVDVRQAIQK.A |
|  |  |  | K.AVPNQLVLGK.M |
| **Others** | | | |
| L63 | gi|168047657 | predicted protein | K.ALQAVVNK.I |
|  |  |  | K.LTLEPNKVHLFLQAADGK.L |
|  |  |  | K.LEPLLEDKEEDEGLTTK.W |
|  |  |  | R.GMQDLQIKLAR.L + Oxidation (M) |
|  |  |  | K.EHETVVRESASYGAR.V |
|  |  |  | K.EMETLSHYTRNHWAR.A |
|  |  |  | R.LREKPLPK.I |
|  |  |  | R.LYDIEGQLRK.I |
|  |  |  | R.SIPITGVGRK.R |
|  |  |  | K.IEEIQRIR.L |
|  |  |  |  |
| L64 | gi|302772723 | hypothetical protein | K.AANDALPKDTPEAK.D |
|  |  |  | R.SRSPEGWAR.R |
|  |  |  | R.LGLVERR.T |
|  |  |  | K.TPQSSSDDTDSKVILVTNVHFATTK.E |
|  |  |  | K.DANVAETSSPAAAVMRPRPPVR.M |
|  |  |  | K.DANVAETSSPAAAVMRPRPPVRMPMR.A |
|  |  |  |  |
| L65 | gi|302823293 | hypothetical protein ELMODRAFT-449095 | K.AANDALPKDTPEAK.D |
|  |  |  | R.SRSPEGWAR.R |
|  |  |  | R.LGLVERR.T |
|  |  |  | R.MRQVELEVTK.L + Oxidation (M) |
|  |  |  | K.TPQSSSDDTDSKVILVTNVHFATTK.E |
|  |  |  | K.EAISTHFGSCGEVVKVTMLTDGVSGKPK.G |
|  |  |  | K.DANVAETSSPAAAVMRPRPPVR.M |
|  |  |  | K.DANVAETSSPAAAVMRPRPPVRMPMR.A |
|  |  |  |  |
| L66 | gi|167998464 | predicted protein | R.MGNESSTIESFSTEFR.R + Oxidation (M) |
|  |  |  | R.MGNESSTIESFSTEFRR.D + Oxidation (M) |
|  |  |  | K.SHLQMYRSGR.I |
|  |  |  | R.SGRINTDGMPK.S + Oxidation (M) |
|  |  |  | K.TGRNSELLR.G |
|  |  |  | R.GTLGNHGFQGHMGEK.S |
|  |  |  | K.GIEFNLTMPNR.S + Oxidation (M) |
|  |  |  |  |
| L67 | gi|297797753 | predicted protein | -.MAMASQVSR.S + 2 Oxidation (M) |
|  |  |  | K.ITRALGR.I |
|  |  |  | K.MMVDEDLELAK.R |
|  |  |  | K.MMVDEDLELAKR.E + Oxidation (M) |
|  |  |  | K.VLADAGYIDAQQQP.- |
|  |  |  |  |
| L68 | gi|147834872 | hypothetical protein VITISV-040309 | R.GIYAYGFEKPSAIQQR.G |
|  |  |  | R.EDQRILSSGVHVVVGTPGR.V |
|  |  |  | R.ILSSGVHVVVGTPGR.V |
|  |  |  | R.ILSSGVHVVVGTPGRVFDMLR.R |
|  |  |  | R.VFDMLR.R |
|  |  |  | R.VFDMLR.R + Oxidation (M) |
|  |  |  | R.QSLRPDYIK.M |
|  |  |  | K.MFVLDEADEMLSR.G |
|  |  |  | K.MFVLDEADEMLSR.G + Oxidation (M) |
|  |  |  | K.DQIYDIFQLLPSK.V |
|  |  |  | K.VQVGVFSATMPPEALEITR.K |
|  |  |  | K.VQVGVFSATMPPEALEITR.K + Oxidation (M) |
|  |  |  | K.QFYVNVDKEEWK.L |
|  |  |  | K.VDWLTDKMR.S + Oxidation (M) |
|  |  |  | R.VLITTDLLAR.G |
|  |  |  | R.MLFDIQR.F |
|  |  |  | R.MLFDIQR.F + Oxidation (M) |
|  |  |  |  |
| L69 | gi|168004878 | predicted protein | M.GEKIVDHEMSFLSSDLICEILNR.V |
|  |  |  | K.IVDHEMSFLSSDLICEILNR.V |
|  |  |  | R.YVFESEACWER.H |
|  |  |  | K.FYANCYPCLSGRGAVR.A |
|  |  |  | R.ALNENMTVSWILINKK.S |
|  |  |  | K.KSGEMANFASWKPLGGLR.H |
|  |  |  | R.LHGLESVELLNRALGCGR.T |
|  |  |  | K.RGSQHCHQMSR.W |
|  |  |  | R.GSQHCHQMSR.W + Oxidation (M) |
|  |  |  |  |
| L70 | gi|168000362 | predicted protein | -.MATFAKPENALK.R |
|  |  |  | K.IMFKYVELCVDMK.K + 2 Oxidation (M) |
|  |  |  | K.YVELCVDMKK.G + Oxidation (M) |
|  |  |  | R.SDRETVTPWFK.F |
|  |  |  | K.FLWETYRTVLEILR.N |
|  |  |  | K.RTPKPQMMAVYYAK.L + 2 Oxidation (M) |
|  |  |  | R.MANILGFNIDAKK.D |
|  |  |  | K.LPNLSDKLSSASPVPEVR.L |
|  |  |  | K.KACSMISPPTVER.S |
|  |  |  | R.FYQQQQEQAIEQSKK.Q |
|  |  |  |  |
| L71 | gi|116779860 | unknown | M.ATLASGKSNVVK.S |
|  |  |  | K.KCLWPAGRPGDSAASAMR.R |
|  |  |  | K.CLWPAGRPGDSAASAMRR.S |
|  |  |  | R.GTGDALRGGSR.K |
|  |  |  | R.VDMPGLGKEDVK.V |
|  |  |  | K.AQMKNGVLK.I |
|  |  |  |  |
| L72 | gi|218201086 | hypothetical protein OsI-29089 | K.LQVSEAEIKALSFSYVAMLK.E |
|  |  |  | K.LREENGSLK.R |
|  |  |  | K.ERISTNMEELHNELSEK.E |
|  |  |  | K.EHVSDASLQSLRSMVMALQK.E |
|  |  |  | R.AHIVQLERALK.F |
|  |  |  | R.LGGDLAMAREELAK.L |
|  |  |  | K.EVLDLMVRMLGFSEEDK.Q + 2 Oxidation (M) |
|  |  |  | K.GVVRGVLGLPGR.L |
|  |  |  |  |
| L73 | gi|49389230 | hypothetical protein | R.SGSSLDARDGGTGLVVPPAR.R |
|  |  |  | R.DPGAPAVVEPPERR.A |
|  |  |  | R.ASPITNPVIHR.G |
|  |  |  | R.GLIYLLDEQGR.L |
|  |  |  | K.IELEWMQNR.K |
|  |  |  | K.IELEWMQNR.K + Oxidation (M) |
|  |  |  | K.VLLAPRCCGT.- |
|  |  |  |  |
| L74 | gi|297721931 | Os03g0229600 | R.RMAASSSLR.L |
|  |  |  | R.MAASSSLR.L |
|  |  |  | R.MAASSSLRLILGSSSASR.R + Oxidation (M) |
|  |  |  | R.KEKPEELVVALAHAK.V |
|  |  |  | K.EKPEELVVALAHAKVTPFLLLLFK.N |

^a^Assigned spot number as indicated in Figure 3 A and Table 1.

^b^Database accession numbers according to NCBInr.

^c^The name of the proteins identified by MALDI-TOF MS.
