# Supplementary material for: Proteomic Analysis Reveals Differences in Tolerance to Acid Rain in Two Broad-Leaf Tree Species, Liquidambar formosana and Schima superba
Source: PLoS One. 2014 Jul 15;9(7):e102532. doi: 10.1371/journal.pone.0102532 (PMC4099204; doi:10.1371/journal.pone.0102532)
Supplement: Table S2 — Details of identified proteins and peptides list of each protein in AR-treated S. superba. (DOC) [file pone.0102532.s003.doc]

**Supplemental Table S2. Details of identified proteins and peptides list of each protein in AR-treated *S. superba.***

| **Spot^a^** | **NCBI accession^b^** | **Protein identity^c^** | **Peptide sequence** |
| --- | --- | --- | --- |
| **Material metabolism** | | | |
| S1 | gi|62321345 | glutamate-ammonia ligase | R.YILER.I |
|  |  |  | R.EEGGYEIIKK.A |
|  |  |  | R.HKEHIAAYGEGNER.R |
|  |  |  | K.EHIAAYGEGNER.R |
|  |  |  | R.GASIRVGR.D |
|  |  |  | K.GYFEDR.R |
|  |  |  |  |
| S2 | gi|205277664 | granule-bound starch synthase I (GBSS) | R.GEGAEQGGATGR.G |
|  |  |  | R.QAGGAEGPRPDDR.R |
|  |  |  | R.SPGHREEEVR.A |
|  |  |  | R.GHEPCGLIQLQGMR.Y + Oxidation (M) |
|  |  |  |  |
| S3 | gi|60101355 | glutamine synthetase (GS) | K.IFSHPDVVAEVPWYGIEQEYTLLQK.D |
|  |  |  | R.YILER.I |
|  |  |  | R.HKEHIAAYGEGNER.R |
|  |  |  | K.EHIAAYGEGNER.R |
|  |  |  | R.HETADINTFLWGVANR.G |
|  |  |  | K.GYFEDR.R |
|  |  |  |  |
| S19 | gi|303280145 | glycosyltransferase family 7 protein | R.RDSANGWAR.S |
|  |  |  | R.ANAWLSEVK.A |
|  |  |  | R.DQLEVFSAYMK.Q + Oxidation (M) |
|  |  |  | K.IYVAEQSNQSAFNRGWALNAAFMNAER.D + Oxidation (M) |
|  |  |  | R.AAPVHTAPRTVILGVNYR.Y |
| **Photosynthesis and energy production** | | | |
| S6 | gi|255544584 | phosphoglycerate kinase (PGK) | -.MASATAPTTLSLLK.T |
|  |  |  | K.VILSSHLGRPK.G |
|  |  |  | K.FSLAPLVPR.L |
|  |  |  | R.LSELLGIQVVK.A |
|  |  |  | K.KLASLADLYVNDAFGTAHR.A |
|  |  |  | K.LASLADLYVNDAFGTAHR.A |
|  |  |  | K.FLKPSVAGFLLQK.E |
|  |  |  | K.ELDYLVGAVSSPKRPFAAIVGGSK.V |
|  |  |  | K.RPFAAIVGGSK.V |
|  |  |  | K.AQGLSVGSSLVEEDKLDLATSLLAK.A |
|  |  |  | K.GVSLLLPTDVVIADK.F |
|  |  |  | K.GVSLLLPTDVVIADKFAPDANSK.V |
|  |  |  | K.VGVAEVMSHISTGGGASLELLEGK.E |
|  |  |  |  |
| S7 | gi|37721507 | photosystem II subunit H | -.MATQTVEGSSR.A |
|  |  |  | M.ATQTVEGSSR.A |
|  |  |  | M.ATQTVEGSSRARPR.Q |
|  |  |  |  |
| S8 | gi|5708095 | ATP synthase gamma chain | K.VALVVVTGDR.G |
|  |  |  | R.GLCGGFNNFIIK.K |
|  |  |  | K.KGNSYFLR.R |
|  |  |  | K.GNSYFLR.R |
|  |  |  | R.RPYIPVDK.Y |
|  |  |  | K.EAQAVADDVFSLFISEEVDKVELLYTK.F |
|  |  |  | K.GEICDINGTCVDAAEDEFFR.L |
|  |  |  | R.ALQESLASELAAR.M |
|  |  |  | K.SLSMVYNR.K |
|  |  |  |  |
| S9 | gi|6688696 | ribulose-1,5-bisphosphate carboxylase/oxygenase large subunit | K.AQAGTGEIKGHYLNATAGTCEEMIK.R + Oxidation (M) |
|  |  |  | R.DNGLLLHIHR.A |
|  |  |  | R.QKNHGMHFR.V + Oxidation (M) |
|  |  |  | K.NHGMHFR.V |
|  |  |  | K.LEGEREITLGFVDLLR.D |
|  |  |  | R.EITLGFVDLLR.D |
|  |  |  | R.VALEACVQAR.N |
|  |  |  | K.WSPELAAACEVWK.E |
|  |  |  | K.EIKFEFPAMDTL.- |
|  |  |  | K.EIKFEFPAMDTL.- + Oxidation (M) |
|  |  |  |  |
| S11 | gi|290490212 | ATP synthase CF1 alpha subunit protein | R.ADEISNIIR.E |
|  |  |  | R.ERIEQYNR.E |
|  |  |  | R.IEQYNR.E |
|  |  |  | R.EVTIVNTGTVLQVGDGIAR.I |
|  |  |  | R.IAQIPVSEAYLGR.V |
|  |  |  | R.VINALAKPIDGR.G |
|  |  |  | R.LIESPAPGIISR.R |
|  |  |  | R.RSVYEPLQTGLIAIDSMIPIGR.G |
|  |  |  | R.SVYEPLQTGLIAIDSMIPIGR.G |
|  |  |  | R.SVYEPLQTGLIAIDSMIPIGR.G + Oxidation (M) |
|  |  |  | R.HTLIIYDDLSK.Q |
|  |  |  | R.QMSLLLR.R |
|  |  |  | R.EAYPGDVFYLHSR.L |
|  |  |  | K.LKLELAQFAELEAFAQFASDLDK.A |
|  |  |  | R.KFLVELR.T |
|  |  |  | K.FLVELR.T |
|  |  |  | K.EAIQEQMER.F |
|  |  |  |  |
| S12 | gi|13430334 | rubisco activase | K.NFMNLPNIK.I + Oxidation (M) |
|  |  |  | K.IPLILGIWGGK.G |
|  |  |  | K.SFQCELVFAK.M |
|  |  |  | K.MCCLFINDLDAGAGR.M |
|  |  |  | R.VPIIVTGNDFSTLYAPLIR.D |
|  |  |  | K.FYWAPTR.D |
|  |  |  | R.VYDDEVRK.W |
|  |  |  | K.LLEYGNMLVQEQENVKR.V |
|  |  |  | K.LLEYGNMLVQEQENVKR.V + Oxidation (M) |
|  |  |  | K.YLSEAALGDANQDAIK.T |
|  |  |  |  |
| S13 | gi|308320553 | ribulose-1,5-bisphosphate carboxylase/oxygenase large subunit | R.QKNHGMHFR.V + Oxidation (M) |
|  |  |  | R.MSGGDHIHSGTVVGK.L |
|  |  |  | R.EITLGFVDLLR.D |
|  |  |  | R.VALEACVQAR.N |
|  |  |  | K.EIKFEFQAMDTLDGDK.D |
|  |  |  |  |
| S14 | gi|170664996 | ribulose-1,5-bisphosphate carboxylase/oxygenase large subunit | K.DTDILAAFR.V |
|  |  |  | K.YGRPLLGCTIKPK.L |
|  |  |  | K.DDENVNSQPFMR.W + Oxidation (M) |
|  |  |  | R.DRFLFCAEALFK.A |
|  |  |  | R.DNGLLLHIHR.A |
|  |  |  | R.QKNHGMHFR.V + Oxidation (M) |
|  |  |  | K.NHGMHFR.V |
|  |  |  | R.MSGGDHIHAGTVVGK.L |
|  |  |  | R.VALEACVQAR.N |
|  |  |  | R.DLACEGNEVIR.E |
|  |  |  | K.EIKFEFQAMD.- |
|  |  |  |  |
| S10 | gi|81301612 | protein Ycf2 | K.KCSMPESNWGSR.W |
|  |  |  | K.ISNETVAGIEILFKEK.D |
|  |  |  | K.DEPDMDSSNKISFLNK.N + Oxidation (M) |
|  |  |  | R.SMNRDPDAYR.Y |
|  |  |  | K.WMIDSFHTR.N + Oxidation (M) |
|  |  |  | K.SFHFPSRSDPFVR.R |
|  |  |  | K.FVSIFHDIMHGSGISWR.I + Oxidation (M) |
|  |  |  | K.LCLPQWNLISEISSK.C |
|  |  |  | R.GSASGGNMLGPAYGVK.S |
|  |  |  | R.IDQILLSLTHSDR.L |
|  |  |  | R.MNGLTMDMMPEIDR.F + 4 Oxidation (M) |
|  |  |  | K.LNTCIK.I |
|  |  |  | K.HFFTLSYTRGFHLEK.K |
|  |  |  | K.MFHTNGFGSITMGSNAR.D |
|  |  |  | K.WYFELGTSMK.R + Oxidation (M) |
|  |  |  | R.TNSSLSNGSFR.S |
|  |  |  |  |
| S20 | gi|303283276 | beta carbonic anhydrase | -.MSAALNDPKIAHVLSGYK.R |
|  |  |  | K.TPLLLVMGHSQCGGCAHALNVVTK.N + Oxidation (M) |
|  |  |  | K.FDPQIRGR.Q |
|  |  |  | K.RVSEYPWVK.D |
|  |  |  | K.MAERVGW.- |
| **Stress and defense** | | | |
| S16 | gi|3328221 | thioredoxin peroxidase (TPx) | K.YVILFFYPLDFTFVCPTEITAFSDR.H |
|  |  |  | K.INTEILGVSVDSVFSHLAWVQTER.K |
|  |  |  | K.INTEILGVSVDSVFSHLAWVQTERK.S |
|  |  |  | K.EGVIQHSTINNLGIGRSVDETLR.T |
|  |  |  | R.TLQALQYVQENPDEVCPAGWKPGEK.S |
|  |  |  |  |
| S17 | gi|2654208 | heat shock 70 | K.MSPSTACIGNPKSAFLR.L |
|  |  |  | K.QFAAEEISAQVLR.K |
|  |  |  | K.QFAAEEISAQVLRK.L |
|  |  |  | K.AVVTVPAYFNDSQR.T |
|  |  |  | R.IAGLEVLR.I |
|  |  |  | R.IINEPTAASLAYGFEK.K |
|  |  |  | R.IINEPTAASLAYGFEKK.N |
|  |  |  | K.MELSSLTQANISLPFITATADGPK.H + Oxidation (M) |
|  |  |  | R.AKFEELCSDLLDR.L |
|  |  |  | K.SEVFSTAADGQTSVEINVLQGER.E |
|  |  |  | K.SEVFSTAADGQTSVEINVLQGEREFVR.D |
|  |  |  | K.KQDITITGASTLPGDEVER.M |
|  |  |  |  |
| S18 | gi|116323 | endochitinase 3 | R.CPSGMCCSNFGWCGNTQDYCGPGK.C + Oxidation (M) |
|  |  |  | K.HRNDNTCQGK.S |
|  |  |  | R.EVAAFFAQTSHETTGGWDTAPDGR.Y |
|  |  |  | K.YYGRGPIQISYNYNYGPCGR.A |
|  |  |  | K.SAIWFWMTAQSPKPSCHDVITGR.W + Oxidation (M) |
|  |  |  |  |
| S21 | gi|384247250 | clavaminate synthase-like protein | M.KLSTTHR.N |
|  |  |  | R.VPGHPMLAVRGK.T + Oxidation (M) |
|  |  |  | K.TSFVPVWHMDK.N |
|  |  |  | K.NELVCPPELCSIYMLK.A + Oxidation (M) |
|  |  |  | K.SIFYNTALFTNFVGYSR.Q |
| **Signal transduction** | | | |
| S15 | gi|350536755 | 14-3-3 protein 4 | K.LAEQAERYEEMIEFMEK.V |
|  |  |  | R.IISSIEQKEESR.G |
|  |  |  | K.SAQDIALAELAPTHPIR.L |
|  |  |  | R.LGLALNFSVFYYEILNSPDR.A |
|  |  |  | K.QAFDEAISELDTLGEESYKDSTLIMQLLR.D |
|  |  |  | K.DSTLIMQLLRDNLTLWTSDNADDVGDDIK.E |
|  |  |  |  |
| S22 | gi|115393868 | phytocyanin-like arabinogalactan-protein (PLA) | K.EIMVGGKTGAWK.I |
|  |  |  | K.EDYTSCNTSNPIAEYKDGNTK.V |
|  |  |  | K.SGPYFFMSGAKGHCEQGQK.M |
|  |  |  | K.SGPYFFMSGAKGHCEQGQK.M |
|  |  |  |  |
| S27 | gi|110532561 | calmodulin (CaM) | M.GMADQLTEDQISEFR.E + Oxidation (M) |
|  |  |  | R.EAFSLFDKDGDGCITTK.E |
|  |  |  | K.LTDEEVDEMIR.E |
|  |  |  | K.LTDEEVDEMIREADVDGDGQINYEEFVK.V |
|  |  |  |  |
| S28 | gi|224131906 | calcium dependent protein kinase 6 (CDPK) | R.GVDNQAYYVLGHK.T |
|  |  |  | R.VIAESLSEEEIAGLKEMFK.A + Oxidation (M) |
|  |  |  | K.EVDQDNDGRIDYGEFVAMMQK.G + 2 Oxidation (M) |
|  |  |  | R.IDYGEFVAMMQK.G + Oxidation (M) |
|  |  |  | R.IDYGEFVAMMQKGNAGIGR.R + 2 Oxidation (M) |
|  |  |  | R.TMRNSLNMSMR.D |
|  |  |  | R.NSLNMSMRDAPGAL.- + Oxidation (M) |
| **Transcription** | | | |
| S23 | gi|108861639 | transposase | K.IMLLTALARPRFDSDGNCTFDGK.I |
|  |  |  | K.IMLLTALARPRFDSDGNCTFDGK.I + Oxidation (M) |
|  |  |  | R.FDSDGNCTFDGKIGCFPFVTYEPAK.R |
|  |  |  | K.RSSANRPAGTIEMKPIESITK.E |
|  |  |  | R.SSANRPAGTIEMKPIESITKEVIR.T |
|  |  |  |  |
| S24 | gi|379041605 | maturase K | K.FQNLR.S |
|  |  |  | K.MEHFWIMYPRFSR.K |
|  |  |  | K.NRWFFMDPLIHYVR.Y |
|  |  |  | K.NRWFFMDPLIHYVR.Y + Oxidation (M) |
|  |  |  | R.WFFMDPLIHYVR.Y |
|  |  |  | R.YQGKAILASK.G |
|  |  |  | K.SPLLVR.N |
|  |  |  | R.NKMLENSFLIDTR.M + Oxidation (M) |
|  |  |  |  |
| S25 | gi|255660958 | pentatricopeptide repeat-containing protein | R.SVSSFNIMMR.G |
|  |  |  | R.ALYVLNGMIK.S + Oxidation (M) |
|  |  |  | K.NEMFGEAYNLVKELLDK.G |
|  |  |  | K.GLDPGVITYSMLMK.G + 2 Oxidation (M) |
|  |  |  | K.GLDPGVITYSMLMKGLCLDHK.V + 2 Oxidation (M) |
|  |  |  | K.GLCLDHK.V |
|  |  |  | K.CAPNLVSHNTLMEGFYK.D |
|  |  |  | K.CAPNLVSHNTLMEGFYK.D + Oxidation (M) |
|  |  |  |  |
| S26 | gi|11993344 | marpoflo protein | R.RESAMMMPESIAPVNALNLNSK.E + Oxidation (M) |
|  |  |  | R.ESAMMMPESIAPVNALNLNSK.E |
|  |  |  | R.ESAMMMPESIAPVNALNLNSK.E + Oxidation (M) |
|  |  |  | R.EHPFIVTEPGEAARGK.K |
|  |  |  | K.ERGENVGAWR.G |
| **Post-translational modification** | | | |
| S4 | gi|225441985 | proteasome subunit alpha type-5 isoform 1 | R.LFQVEYAIEAIK.L |
|  |  |  | K.IMEIDEHIGCAMSGLIADAR.T |
|  |  |  | R.FSYGEPMTVESTTQALCDLALR.F |
|  |  |  | R.FSYGEPMTVESTTQALCDLALR.F + Oxidation (M) |
|  |  |  | K.VAPTYHLYTPAEVEAVISR.L |
|  |  |  | K.VAPTYHLYTPAEVEAVISRL.- |
|  |  |  |  |
| S5 | gi|356545337 | mitochondrial import receptor subunit TOM6 homolog isoform 1 | -.MFPGMFMRKPDK.A + 2 Oxidation (M) |
|  |  |  | M.FPGMFMRKPDK.A + Oxidation (M) |
|  |  |  | K.AAALKQLK.S |
|  |  |  | K.QLKSHAAMFGTWVVVIR.V |
| **Others** | | | |
| S29 | gi|224064392 | predicted protein | K.INHETNKPMFTTLFTHMKNLDR.R + 2 Oxidation (M) |
|  |  |  | K.LLLSLDLDDPMGAMFCVDYFALR.A + Oxidation (M) |
|  |  |  | R.AEEYAWLEWFSEDYK.S |
|  |  |  | K.DDDTCATKSSSADLMK.Q + Oxidation (M) |
|  |  |  | K.QALMLHPSVLK.K |
|  |  |  | K.LLRNSALQVIETLEHSSSDAK.D |
|  |  |  | R.KEAFSSENNE.- |
|  |  |  |  |
| S30 | gi|388496926 | unknown | R.KLQSDELATVR.L |
|  |  |  | R.LFQENTPSVVYITNLAVK.Q |
|  |  |  | K.DVAVLRVDAPK.D |
|  |  |  | K.DKLRPIPVGVSADLLVGQK.V |
|  |  |  | K.LRPIPVGVSADLLVGQK.V |
|  |  |  | K.VYAIGNPFGLDHTLTTGVISGLR.R |
|  |  |  | K.VYAIGNPFGLDHTLTTGVISGLRR.E |
|  |  |  | K.VTRPILGIK.F |
|  |  |  |  |
| S31 | gi|242052501 | hypothetical protein SORBIDRAFT_03g010120 | -.MGANCCVAAKER.T |
|  |  |  | R.GTSPSNVFHGAK.W |
|  |  |  | R.KSLDMSSVASDLK.T |
|  |  |  | K.SLDMSSVASDLK.T |
|  |  |  | R.QVSDSKIPSLR.S |
|  |  |  | R.TFSEMVATSQR.E |
|  |  |  | R.SSASNSTTLPPDQEVCK.L |
|  |  |  | R.EPRLGTSSSMK.V |
|  |  |  | R.HFSIGSRPPR.S |
|  |  |  |  |
| S32 | gi|168071263 | predicted protein | R.EVRVVGDPIGIIGMGPGGQMHK.Q + Oxidation (M) |
|  |  |  | R.FDKPLQVGAVFPAGSR.I |
|  |  |  | R.IFLLLIVVAELDEHVVAR.L |
|  |  |  | R.IFLLLIVVAELDEHVVARLK.L |
|  |  |  |  |
| S33 | gi|77554095 | hypothetical protein LOC_Os12g13240 | R.NGIASAVPPVPMESR.V + Oxidation (M) |
|  |  |  | R.THPSDGNQAPTR.T |
|  |  |  | K.TANHSLMDYK.V + Oxidation (M) |
|  |  |  | R.STSQEDMSSRAAK.S + Oxidation (M) |
|  |  |  | R.SNNDEFKLEALQR.N |
|  |  |  | R.FYTARMIK.S |
|  |  |  |  |
| S34 | gi|145347277 | predicted protein | R.GAMPAPADAKR.V |
|  |  |  | K.LALGADGTKK.Q |
|  |  |  | R.QATETLGDSALDDDSIK.E |
|  |  |  | K.YYLHSLGDYFKMESDSQK.F + Oxidation (M) |
|  |  |  | K.ETTATQTIGDDGSK.A |
|  |  |  | R.GIGDDIDLLVEEAVECAGK.E |
|  |  |  | K.LEQIRENFR.K |

^a^Assigned spot number as indicated in Figure 3 C and Table 2.

^b^Database accession numbers according to NCBInr.

^c^The name of the proteins identified by MALDI-TOF MS.
